# New constraints of terrestrial and oceanic global gross primary productions from the triple oxygen isotopic composition of atmospheric CO_2_ and O_2_

**DOI:** 10.1038/s41598-023-29389-z

**Published:** 2023-02-07

**Authors:** Mao-Chang Liang, Amzad H. Laskar, Eugeni Barkan, Sally Newman, Mark H. Thiemens, Ravi Rangarajan

**Affiliations:** 1 Institute of Earth Sciences, Academia Sinica, Taipei, Taiwan; 2 Physical Research Laboratory, Ahmedabad, Gujarat India; 3 Institute of Earth Sciences, Hebrew University of Jerusalem, Jerusalem, Israel; 4 Division of Geological and Planetary Science, California Institute of Technology, Pasadena, CA USA; 5 Department of Chemistry and Biochemistry, University of California at San Diego, La Jolla, CA USA; 6 Present Address: Bay Area Air Quality Management District, San Francisco, USA; 7 Present Address: Department of Public Health, College of Health Sciences, University of Doha for Science and Technology, Doha, Qatar

**Keywords:** Biogeochemistry, Environmental sciences

## Abstract

Representations of the changing global carbon cycle under climatic and environmental perturbations require highly detailed accounting of all atmosphere and biosphere exchange. These fluxes remain unsatisfactory, as a consequence of only having data with limited spatiotemporal coverage and precision, which restrict accurate assessments. Through the nature of intimate coupling of global carbon and oxygen cycles via O_2_ and CO_2_ and their unique triple oxygen isotope compositions in the biosphere and atmosphere, greater insight is available. We report analysis of their isotopic compositions with the widest geographical and temporal coverage (123 new measurements for CO_2_) and constrain, on an annual basis, the global CO_2_ recycling time (1.5 ± 0.2 year) and gross primary productivities of terrestrial (~ 170–200 PgC/year) and oceanic (~ 90–120 PgC/year) biospheres. Observed inter-annual variations in CO_2_ triple oxygen isotopic compositions were observed at a magnitude close to the largest contrast set by the terrestrial and oceanic biospheres. The seasonal cycles between the east and west Pacific Ocean were found to be drastically different. This intra-annual variability implies that the entire atmospheric CO_2_ turnover time is not much longer than the tropospheric mixing time (less than ~ 5 months), verifying the derived recycling time. The new measurements, analyses, and incorporation of other global data sets allow development of an independent approach, providing a strong constraint to biogeochemical models.

## Introduction

Imbalance between CO_2_ sources and sinks results in increasing atmospheric CO_2_ levels^1–3^. The increase is due mainly to fossil fuel burning, emitting at an average rate of 9.4 ± 0.5 PgC/year^2^. As a consequence, the average global temperature has increased by 1.1 °C^3^. The combined effect of elevated atmospheric CO_2_ levels and temperature leads to observable changes in the global carbon cycle^4,5^. A number of efforts have been used to quantify the changes of the carbon cycles in response to changing environments^1,3,4,6–8^. Climate models coupled with biogeochemical modules are frequently used to assess changing ecosystems in the context of changing climate^1,3,8,9^. However, it has been noted that current knowledge is insufficient to simulate and project the ecosystem responses^6,7^, owing to the boundaries of our knowledge of the gross processes. Reviewing the input components and processes considered^9–11^, terrestrial net ecosystem exchange (NEE) is better studied but gross primary production (GPP) processes (such as photosynthesis) comprising > 3/4 of the total carbon fluxes in the carbon cycling budget remain least well-constrained^8,10–18^. The incomplete knowledge of the gross processes is largely due to rapid hydration and dehydration of CO_2_ occurring in chloroplasts; it is also because of this fast reaction that regional and global assessments of the gross components are possible^11,15,17,19^. The carbonic anhydrase catalyzed process is, however, modulated by the impact of hydrological cycles and evapotranspiration, creating spatiotemporal inhomogeneities and variability. These complexities restrict carbon cycle quantification. Although regional and local carbon cycling fluxes have been extensively evaluated^13^, the magnitudes of the gross fluxes, including the global assessment extrapolated from regional/local measurements, remain inconclusive^8,10–13,15–18,20^.

In addition to applying a commonly utilized eddy-covariance method to terrestrial net ecosystem production^13^ for approximating gross components in carbon cycle models^10,11,21,22^ at local and regional scales, global assessment has extensively relied on oxygen isotopic analysis, which, however, is often complicated by source water isotopic inhomogeneity and meteorological dynamics^23^. Examining all the components in biogeochemical models, water isotopic composition in the hydration/dehydration reaction center, where carbonic anhydrase resides, is by far the least well understood, due primarily to water evaporation. As a result, differing interpretations for the biological carbon cycling vary and remain controversial^11,13,15–18,20^. Triple-oxygen isotopic analysis tackles the gross processes from a different vantage point, because of its sensitivity to and conservation in the canonical terrestrial processes, including the aforementioned evaporation^17,24–28^. Oxygen has three stable isotopes (^16^O, ^17^O, and ^18^O). In the present study, we use a linear form for the excess, Δ^17^O, for the carbon cycling flux calculation, because the excess in a typical log definition is not a conserved quantity:

1 $$\Delta^{{{17}}} {\text{O }} = {\updelta }^{{{17}}} {\text{O }} - {\uplambda } \times {\updelta }^{{{18}}} {\text{O}}$$

where the δs are the isotopic compositions of the species of interest, referenced to the VSMOW standard. The core reason for choosing triple oxygen isotopic analysis is that typical biogeochemical processes that modify δ^17^O and δ^18^O follow well-defined mass fractionation slopes in a three-isotope plot, with values close to 0.5^29–31^. The formulation of the carbon flux budget using Δ^17^O is thus simplified, compared to that of δ^18^O, because the non-zero values are not affected by uptake and the uncertainties from water isotopic variability are removed. Multi-isotope measurements have proven valuable in global studies. This work enhances these efforts with new integrative interpretation and modeling focusing on unification of the multiple isotopic systems, in order to provide constraints on narrowing the range of terrestrial gross primary production, tGPP (~ 110–150 PgC/year) from climate models^1,6,8,9^ and allow better resolution of the previously poorly known oceanic production, oGPP^32,33^.

The value of λ, independent of source water isotopic composition, unlike δ, is process-specific and insensitive to temperature^30^. To our knowledge, λ depends on ambient air relative humidity only^34,35^, that largely removes the complexities from water evapotranspiration and equilibrium and kinetics processes associated with water-mediated gross processes. The adopted linear definition follows the same budget formulation as δ^18^O^11,17^ and has been used widely for dissolved O_2_ for assessing aquatic GPP^24–26^. It is recommended that a λ value of 0.516 is best for describing the O_2_ system^25^, same as our preferred choice for the CO_2_ system described below; we also show in the Supplementary Information that the choice only weakly affects the gross fluxes derived in this paper. For atmospheric CO_2_ in the terrestrial biosphere, leaf and soil water is responsible for the oxygen isotopic composition of CO_2_, readily affected by frequent isotope exchange between CO_2_ and water. The oxygen isotopic variation of leaf and soil water is controlled by evapotranspiration^35^, influenced by atmospheric relative humidity. At the globally averaged relative humidity of 75 ± 5%^36^, the value of λ is 0.5160 ± 0.0004^35^. We thus adopt the value of 0.516 for λ for CO_2_, unifying the selection for the O_2_ and CO_2_ systems. (We stress that the selection of the λ value does not change the results (within error) presented in this work. For example, the recycling time derived at λ = 0.516 is 1.5 ± 0.2 year (see below) and it remains at 1.5 year for λ = 0.528 but with a larger error of 0.4 year. This is because the selection does not best represent the variation of tropospheric CO_2_). The co-variation and closure of CO_2_ and O_2_^26,32,33,37^, specifically their triple-oxygen isotope compositions, thus, allow us to constrain the global carbon cycles (gross components) from a new and independent perspective, the basis for this new work.

**Figure 1:**
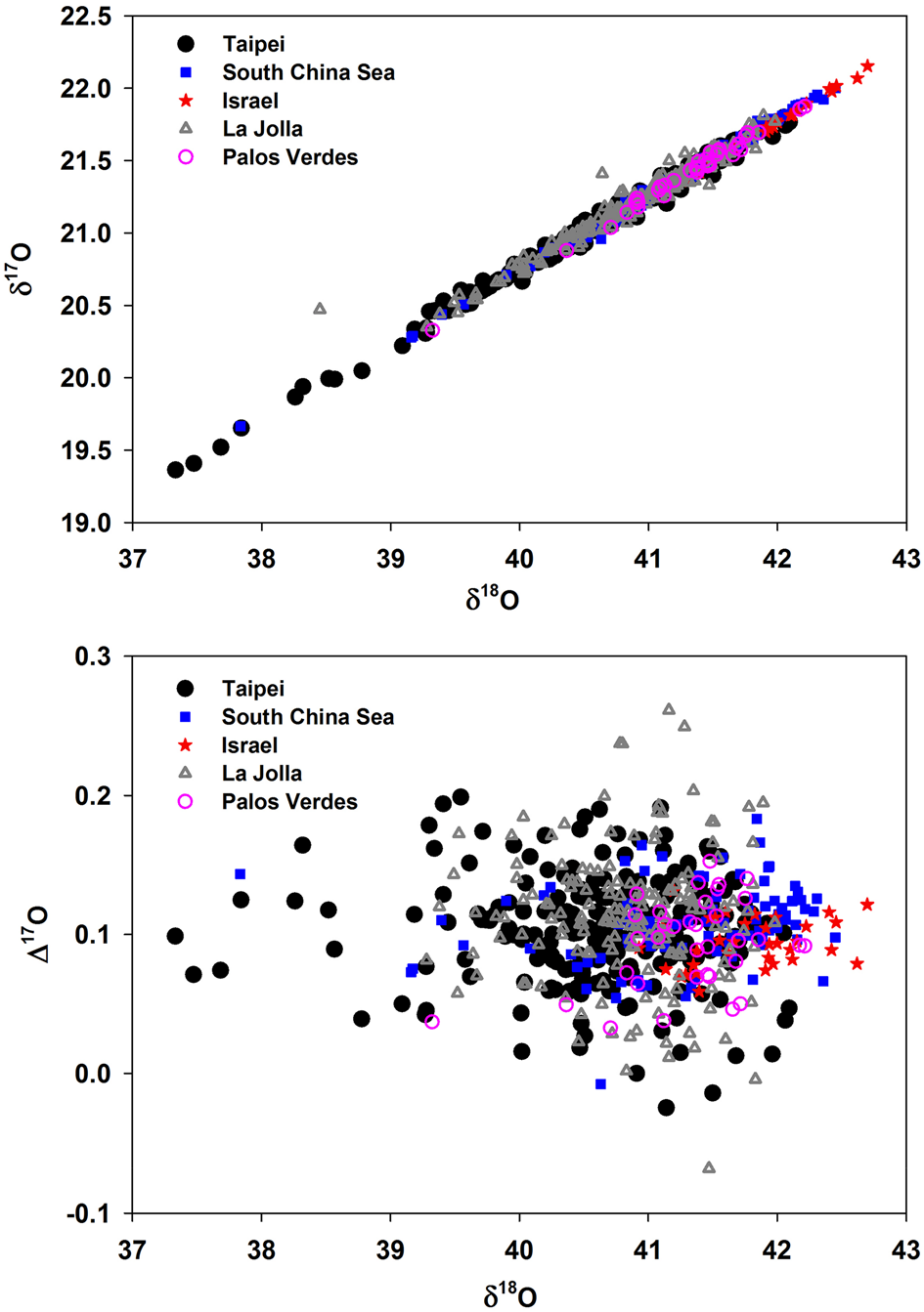
Top: δ^17^O vs. δ^18^O plot for atmospheric CO_2_ collected from Taipei (Taiwan), South China Sea, Jerusalem (Israel), La Jolla (United States), and Palos Verdes (United States). Values in ‰ are referenced to VSMOW. Bottom: The reported Δ^17^O values. External measurement uncertainty is ~ 0.05‰ for δ^17^O and δ^18^O and ~ 0.01‰ or less for Δ^17^O. The two anomalous points (two triangles well above the others in the top panel) from La Jolla are beyond the plotting range of Δ^17^O and not shown. See Table 1 for the sources of the data. Note that the δ^17^O values have been rescaled from Liang et al.^17^ See text for details.

Two recent attempts were made to provide new insight into the assessment of carbon cycling fluxes from a global perspective. These two approaches are the impact of hydrological cycles affected by ENSO using the δ^18^O values of atmospheric CO_2_ and the extension of single delta values to triple oxygen isotopic analysis. The values of tGPP from the two methods are ~ 150–175^15^ and 120 ± 30^17^ PgC/year, respectively. The merits of the two are different. The former utilizes the changing isotopic signal in precipitation and the quasi-equilibrium isotopic exchange between CO_2_ and rainwater; a global and extensive dataset of surface water is needed to account for spatiotemporal inhomogeneity of precipitation in response to ENSO. The second approach largely reduces the complexity of the analysis, because most of the known biogeochemical processes follow well-defined relations with the change in δ^17^O being about one-half of δ^18^O. Here, we examine the reported GPP values using the latter approach making use of the Δ^17^O values in atmospheric CO_2_ and O_2_, using data with by far the widest spatial and temporal coverage. In comparing and interpreting O_2_ gross production and CO_2_ photosynthetic uptake, we apply a steady state approximation where production of one O_2_ molecule consumes one CO_2_ molecule. The data used in this work are shown in Fig. 1 and summarized in Table 1.

**Table 1:** Δ^17^O values used in the work for the oxygen isotope recycling time and GPP derivations.

	Δ^17^O (‰)	^17^Δ (‰)	Notes (references)
Photosynthetic O_2_	0.201 ± 0.011	0.254 ± 0.011	^25^
Terrestrial O_2_	0.149 ± 0.016	0.242 ± 0.016	^25,52^
Oceanic O_2_	0.202 ± 0.015	0.253 ± 0.015	^25,52^
Meteoric water (n = 40)	− 0.052 ± 0.006	− 0.046 ± 0.005	^52^
Oceanic water (n = 38)	0.000 ± 0.001	0.000 ± 0.001	^52^
Leaf CO_2_	− 0.009 ± 0.006	0.244 ± 0.005	^17^
Soil CO_2_	0.019 ± 0.006	0.244 ± 0.005	^17^
Oceanic CO_2_	0.075 ± 0.001	0.284 ± 0.001	^17^
Anthropogenic CO_2_	− 0.286 ± 0.001	− 0.210 ± 0.001	^17^
AS CO_2_ (n = 146)	0.102 ± 0.003	0.298 ± 0.003	2012.12 ~ 2015.12^17^
NTU CO_2_ (n = 89)	0.102 ± 0.004	0.297 ± 0.004	2013.11 ~ 2015.12^17^
SCS CO_2_ (n = 94)	0.102 ± 0.006	0.305 ± 0.005	2013.06 ~ 2017.11 (^17^; this work)
Israel CO_2_ (n = 34)	0.095 ± 0.003	0.302 ± 0.003	2012.02 ~ 2015.05 (^31^; this work)
La Jolla CO_2_ (n = 180)	0.116 ± 0.005	0.314 ± 0.005	1990.12 ~ 2000.03 (^16^)
Palos Verdes CO_2_ (n = 35)	0.094 ± 0.005	0.297 ± 0.006	2015.04 ~ 2016.03 (^17^; this work)
Location mean for tropospheric CO_2_ ± 1 SE	0.099 ± 0.003	0.301 ± 0.002	Averages of Taiwan (AS + NTU), SCS, Israel, USA (La Jolla + Palos Verdes)

^17^Δ in logarithmic scale is defined as ^17^Δ = ln(1 + δ^17^O) – 0.516 × ln(1 + δ^18^O). O_2_ values are reported referenced to atmospheric O_2_, and CO_2_ are referenced to VSMOW. Photosynthetic O_2_ refers to photosynthesis from VSMOW, terrestrial O_2_ the photosynthesis from the terrestrial biosphere with the Δ^17^O (and ^17^Δ) assumed to be the same as that in meteoric water, and oceanic O_2_ the O_2_ from the oceanic biosphere. Leaf, soil, and oceanic CO_2_ are the CO_2_ in equilibrium with the respective water, taken from Table 1 of Liang et al.^17^.

## Materials and methods

### Air sampling

In addition to using data available in the literature from the middle East^31^, Pacific^16,17^, and South China Sea^17^ regions, we have extended and collected air for isotopic analysis of CO_2_ in four locations: (1) Academia Sinica campus (abbreviated AS; 121°36′51ʺE, 25°02′27ʺN; ~ 10 m above ground level or 60 m above sea level) in Taipei, Taiwan, (2) the campus of National Taiwan University (NTU; 121°32′21ʺE, 25°00′53ʺN; ~ 10 m above ground level or 20 m above sea level; ~ 10 km southwest of Academia Sinica), (3) the southern California coast on Palos Verdes peninsula (118°10.9′W, 33° 44.7′N; PVD), and (4) on the roof of the building of the Institute of Earth Sciences at the Edmond J. Safra campus of Hebrew University in Jerusalem, Israel (35°11′60.00ʺE, 31°46′19.79ʺN; ~ 18 m above ground level or 770 m above sea level).

Air from western Pacific regions was collected for isotopic analysis in pre-conditioned 1-L Pyrex bottles, achieved by passing dry, high purity nitrogen through the bottles overnight. The sampling bottles used for concentration (~ 350-mL bottle) and isotope (1-L) analyses were connected in series. Samples were collected and compressed to 2-bar after flushing the bottles for 5 min with ambient air at a flow rate of ~ 2 L per min. Moisture was removed during sampling using magnesium perchlorate to minimize secondary isotopic exchange between CO_2_ and water. The PVD samples were collected on Saturday afternoons at about 14:00 PST, into 2-L evacuated Pyrex flasks after passing through Mg(ClO_4_)_2_. Carbon dioxide was separated from the air samples cryogenically and measured, following the method described previously^17^. In brief, for samples collected in Taiwan, CO_2_ was extracted by pumping the air at a flow rate of ~ 90 mL/min from the flasks through a series of four coil traps, with first two kept in dry ice-ethyl alcohol slush (− 78 °C) for moisture removal and the others in liquid nitrogen (− 196 °C). For CO_2_ from PVD, it was extracted from the air samples on a glass vacuum line by freezing in liquid nitrogen U-traps containing glass beads, followed by drying in ethanol-dry ice trap.

In Israel, atmospheric air samples were collected in evacuated 5 L flasks, followed by CO_2_ extraction using Russian doll traps according to Brenninkmeijer and Röckmann^38^. See Barkan and Luz^31^ for details.

### Laboratory measurements

Full analytical procedures are described in detail elsewhere^17^ and summarized here. The concentration of CO_2_ is measured using a LI-COR infrared gas analyzer (model 840A, LI-COR, USA), with reproducibility better than 1 ppmv. The CO_2_–O_2_ oxygen isotope exchange method was used to measure the Δ^17^O of CO_2_ samples. Isotopic analyses were done using a FINNIGAN MAT 253 mass spectrometer in dual inlet mode. The analytical precision obtained for a single measurement of the Δ^17^O value of CO_2_ is better than 0.01‰ (1 − σ standard deviation).

In Israel, the measurements of three oxygen isotopes in CO_2_ were carried out by CO_2_ isotopic exchange with O_2_ of known isotopic composition over hot platinum^39^. After isotopic exchange, δ^17^O and δ^18^O of O_2_ were measured in dual-inlet mode by a multi-collector mass spectrometer (Delta Plus, ThermoFisher Scientific, Bremen, Germany). The analytical errors in δ^17^O and δ^18^O are 0.008 and 0.004‰, respectively. All measurements were performed against an in-house CO_2_ standard analyzed daily to determine the performance of the CO_2_–O_2_ isotopic exchange line and the mass spectrometer. See Barkan et al.^39^ for details.

In total, 123 new measurements of the triple oxygen isotope compositions in atmospheric CO_2_ were obtained. Along with the available data from our previous work^16,17^, there are 578 used for deriving the CO_2_ oxygen isotope turnover time and gross primary production of the terrestrial biosphere.

### Inter-calibration of the CO_2_ Δ^17^O scale

New calibrations presented below show that the Δ^17^O values reported by Liang et al.^17^ were biased too high by ~ 0.03–0.04‰. This conclusion results from comparing exchanged aliquots of working CO_2_ gas with water-equilibrated CO_2_. The latter is a process largely controlling the oxygen delta values of CO_2_ (both Δ^17^O and δ^18^O) in the biosphere, providing a robust approach (in contrast to the previous graphite method^40^ for Δ^17^O standardization) to consolidating the scale of Δ^17^O in CO_2_. As a result of the reduced Δ^17^O values in atmospheric CO_2_, the new calibration is expected to yield a shorter CO_2_ recycling time and larger terrestrial carbon cycling flux than those derived previously^17^, as obtained in the main text (1.5 ± 0.2 years here as opposed to 1.9 ± 0.3 years in Liang et al.^17^).

For water-equilibrated CO_2_, we followed the same procedure as earlier^41^ for equilibrating CO_2_ with VSMOW water on a shaking stage at an oscillation frequency of 1 s^−1^ in a thermostatic water bath maintained at 25 °C. About 150-µL of water were introduced to a quarter-inch diameter, 15-cm long Pyrex tube, followed by freezing at acetone-dry ice slush temperature for air evacuation. After evacuation, about 100–150 µmoles of CO_2_ were injected, and the tube was then flamed-sealed. This procedure resulted in the H_2_O:CO_2_ molar ratio being ~ 70. The CO_2_ used was taken from our high purity (> 99.9999%; Air Products, Inc.) CO_2_ cylinder (AS-2) with nominal values of − 32.62‰ (VPDB) and 36.64‰ (VSMOW) for δ^13^C and δ^18^O^42^, respectively. One may question whether this H_2_O:CO_2_ molar ratio introduces noticeable errors in the final determination of the delta values of CO_2_. A mass-balance calculation shows that the ratio affects the δ^18^O values of CO_2_ by as much as ~ 0.2‰. So, for example, a 0.001 shift in λ results in offsetting Δ^17^O by 0.0002‰, negligible compared to the nominal precision of 0.01‰.

The equilibrated CO_2_ was measured for its triple oxygen isotopic composition, following our standard procedure utilizing the technique of CO_2_–O_2_ isotopic exchange over hot platinum^17,40^. The δ^17^O scale was maintained using our working CO_2_ (AS-2) with a nominal value of ^17^Δ = 0.161‰, calibrated against well calibrated AS-1^17,43^. With the established scale for δ^18^O and δ^17^O, we measured VSMOW–equilibrated CO_2_ at 25 °C and the results are summarized in Table S1. The resulting fractionation factors, ^17^α and ^18^α, are 1.02139 ± 0.00001 and 1.04122 ± 0.00002 (standard errors with n = 9), respectively, for ^17^O and ^18^O. The value of ^18^α is about 0.0002 different from Barkan and Luz^31^ or ~ 0.2‰ in the δ^18^O value that, if attributable to temperature, is 1 °C error of the thermostat (our nominal precision). With this, the calculated ln(^17^α)/ln(^18^α) value is 0.52393 ± 0.00009, 0.001 ± 0.0001 higher than that (0.5229 ± 0.0001) from Barkan and Luz^31^. Calculation shows that this shift in the λ value introduces a difference of 0.042 ± 0.006‰ (= (0.52393–0.5229) × 41.2‰, where 41.2‰ is the value of ^18^α − 1 at 25 °C) in Δ^17^O values between the two labs.

Additionally, aliquots of AS-2 CO_2_ were shared with and measured by co-author Barkan in Israel; the measured values are summarized in Table S2. There is a difference of 0.032 ± 0.001‰ in Δ^17^O, consistent with the value noted above from the measurements of water-equilibrated CO_2_. There are two ways to circumvent the issues of Δ^17^O scale. One is to normalize and rescale the measured Δ^17^O values of samples and report the values with respect to the VSMOW-equilibrated CO_2_. The other is to take the value of water-CO_2_ λ from Barkan and Luz^31^ and rescale our measured Δ^17^O values for samples using the two AS-2 values (AS-2 and VSMOW-water-equilibrated AS-2) reported by the two labs. We take the latter approach and rescale the Δ^17^O values with the mean value of 0.037‰ (mean of 0.032‰ and 0.042‰). The tropospheric CO_2_ Δ^17^O values reported in the main text and Supplementary table (Table S3) reflect this scale recalibration.

### Box modeling and gross primary production assessment

For global gross production assessments, we ensured that the length of the sampling for each location is at least one year, to best remove seasonal variations. PVD at the eastern border of the Pacific, the site with the shortest time span of sampling (1 year), though sampling rather clean marine air during the 2014–2016 El Nino, on average, does not show a significant difference (Table 1) in CO_2_ Δ^17^O values relative to the data from the other localities with multi-year data records averaged. Though the mean is the same as the others, the site, however, exhibit statistically significant intra-annual variabilities, shown also from the data at the western side of the Pacific at AS (see below). This new finding poses a new challenge to the current carbon cycling framework, and we will discuss this new issue.

**Figure 2:**
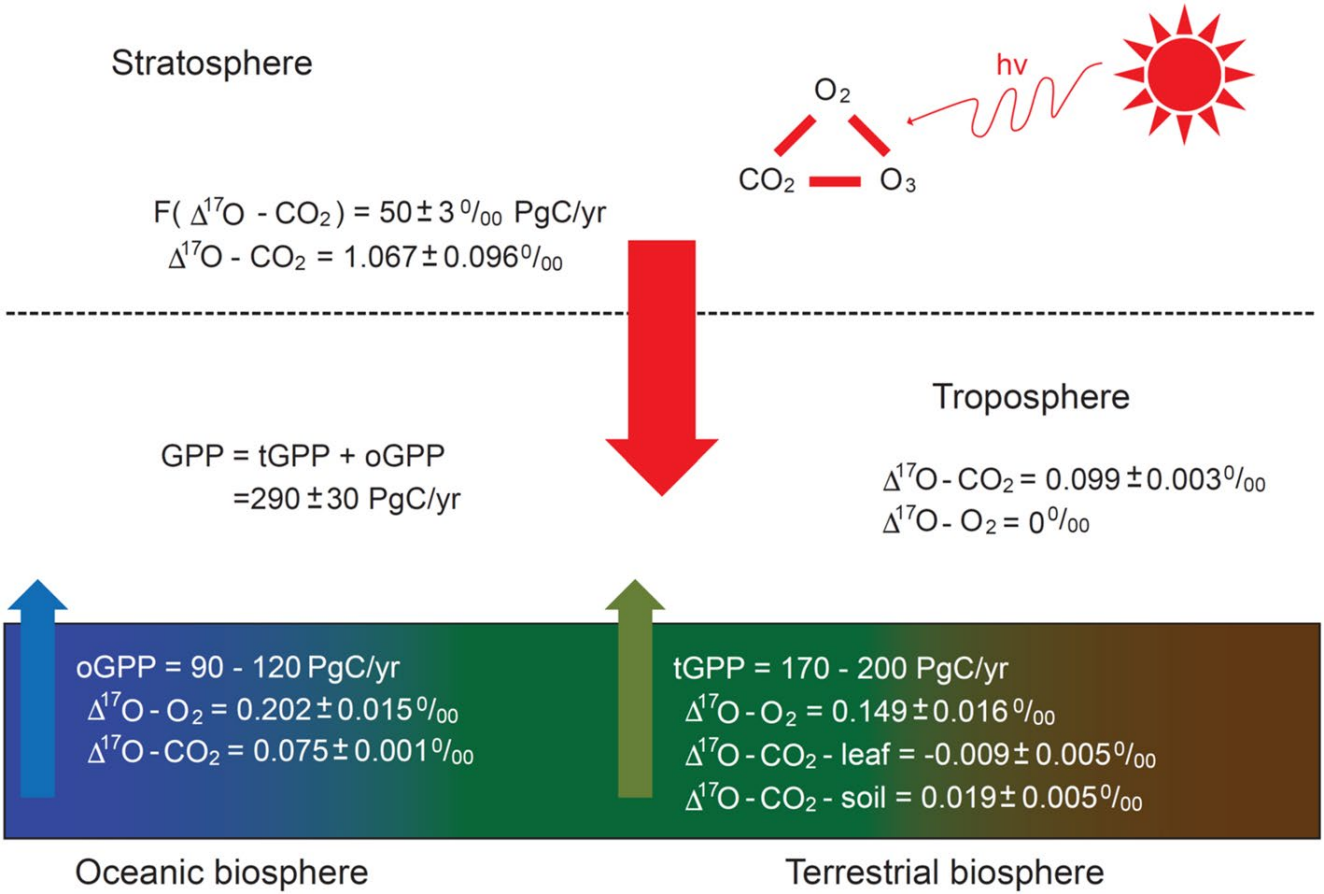
Summary of the budgets of Δ^17^O transport for atmospheric CO_2_ and O_2_ and gross primary productivities (GPP, tGPP, and oGPP) derived in this work.

To utilize Δ^17^O for a gross flux study, one requires at least one source of Δ^17^O that is well-understood and distinct from that of the biosphere and hydrosphere. Stratospheric O_2_–O_3_–CO_2_ photochemistry is the only process that is known to produce large non-zero Δ^17^O values in O_2_ and CO_2_ different from those originating at the surface. Reactions with ozone, as the intermediate, repartition the oxygen isotopes between O_2_ and CO_2_. As a result of the coupled photochemistry, Δ^17^O in stratospheric CO_2_ is enhanced, materially balanced by its depletion in O_2_^44^. When the CO_2_ and O_2_ molecules return to the troposphere, the excess is diluted by various biological and hydrospheric processes, reflected in the value of Δ^17^O in the tropospheric CO_2_ and O_2_^16,17,26^. See Fig. 2 for a schematic diagram of these processes.

**Figure 3:**
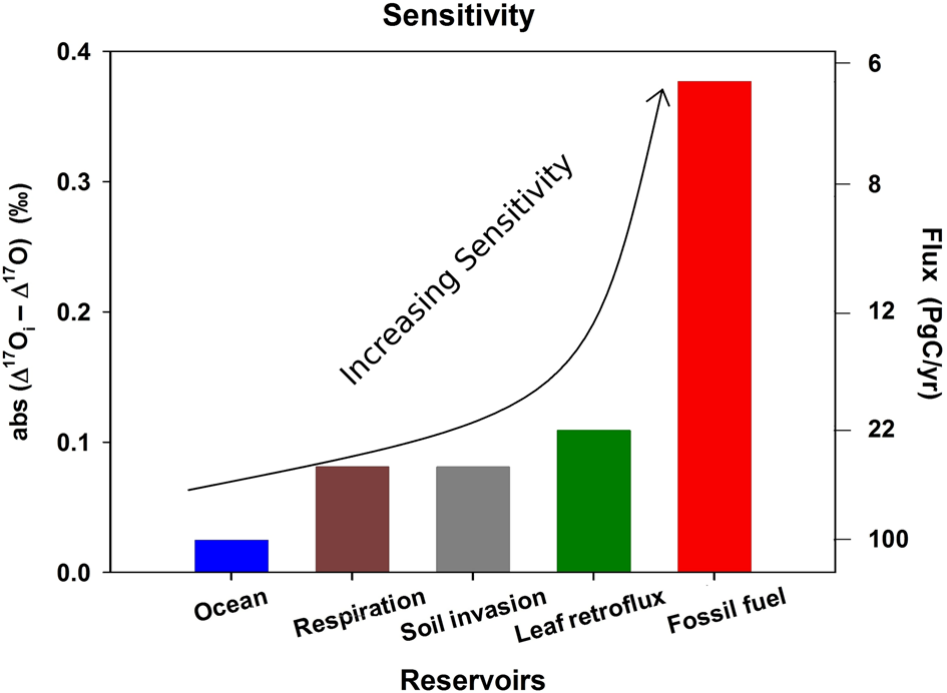
Sensitivity of the processes considered (in terms Δ^17^O_i_; see text and Table 1 for the respective values) as affecting the Δ^17^O budget of CO_2_ in the atmosphere. The corresponding gross fluxes needed are shown on the right-axis; the values are normalized to oceanic flux at 100 PgC/year. The higher the abs (Δ^17^O_i_ − Δ^17^O) value the higher the sensitivity in the global carbon cycling budget. Though the sensitivity to fossil fuel burning is the highest, the flux is lowest and is well-constrained^2^, giving its Δ^17^O isoflux the least significance^17^.

The global carbon cycling budget, at steady state, can be formulated, with respect to CO_2_ and O_2_ in the troposphere (Δ^17^O), as follows:

2 $$\sum_{\mathrm{i}}{\mathrm{F}}_{\mathrm{i}}\times ({\Delta }^{17}{\mathrm{O}}_{\mathrm{i}}-{\Delta }^{17}\mathrm{O})=0$$

where F_i_ is the flux for each reservoir “i" considered, with its characteristic Δ^17^O_i_. For CO_2_, the reservoirs include water equilibrated CO_2_ coming from leaf stomata, soil respiration, soil invasion, and oceans, and CO_2_ from anthropogenic emissions and the stratosphere. Given the sensitivity of the isoflux to the terrestrial CO_2_ processes (Fig. 3; see also Fig. 4 of Liang et al.^17^), the approach would successfully give the flux from the terrestrial biosphere, a poorly constrained quantity in current carbon cycle models^8^. The tGPP may then be determined. Because of its long lifetime in the atmosphere, we use atmospheric O_2_ for the globally averaged GPP, obtained in steady state by balancing the stratospheric O_2_ flux having a negative ^17^O-excess (i.e., Δ^17^O_st_–Δ^17^O < 0) with the positive biospheric O_2_. That is,

3 $${\text{tGPP}} \times (\Delta^{{{17}}} {\text{O}}_{{\text{t}}} - \Delta^{{{17}}} {\text{O}}) \, + {\text{ oGPP}} \times (\Delta^{{{17}}} {\text{O}}_{{\text{o}}} - \Delta^{{{17}}} {\text{O}}) \, + {\text{ F}}_{{{\text{st}}}} \times (\Delta^{{{17}}} {\text{O}}_{{{\text{st}}}} - \Delta^{{{17}}} {\text{O}}) \, = \, 0,$$

following the approach of Luz et al.^26^, with the values of Δ^17^O for atmospheric O_2_, Δ^17^O_t_ for terrestrial photosynthetic O_2_, and Δ^17^O_o_ for oceanic given in Table 1. With the values of GPP and tGPP determined, the oGPP can be calculated (Supplementary Information). We describe below first the tGPP, followed by GPP and oGPP.

**Figure 4:**
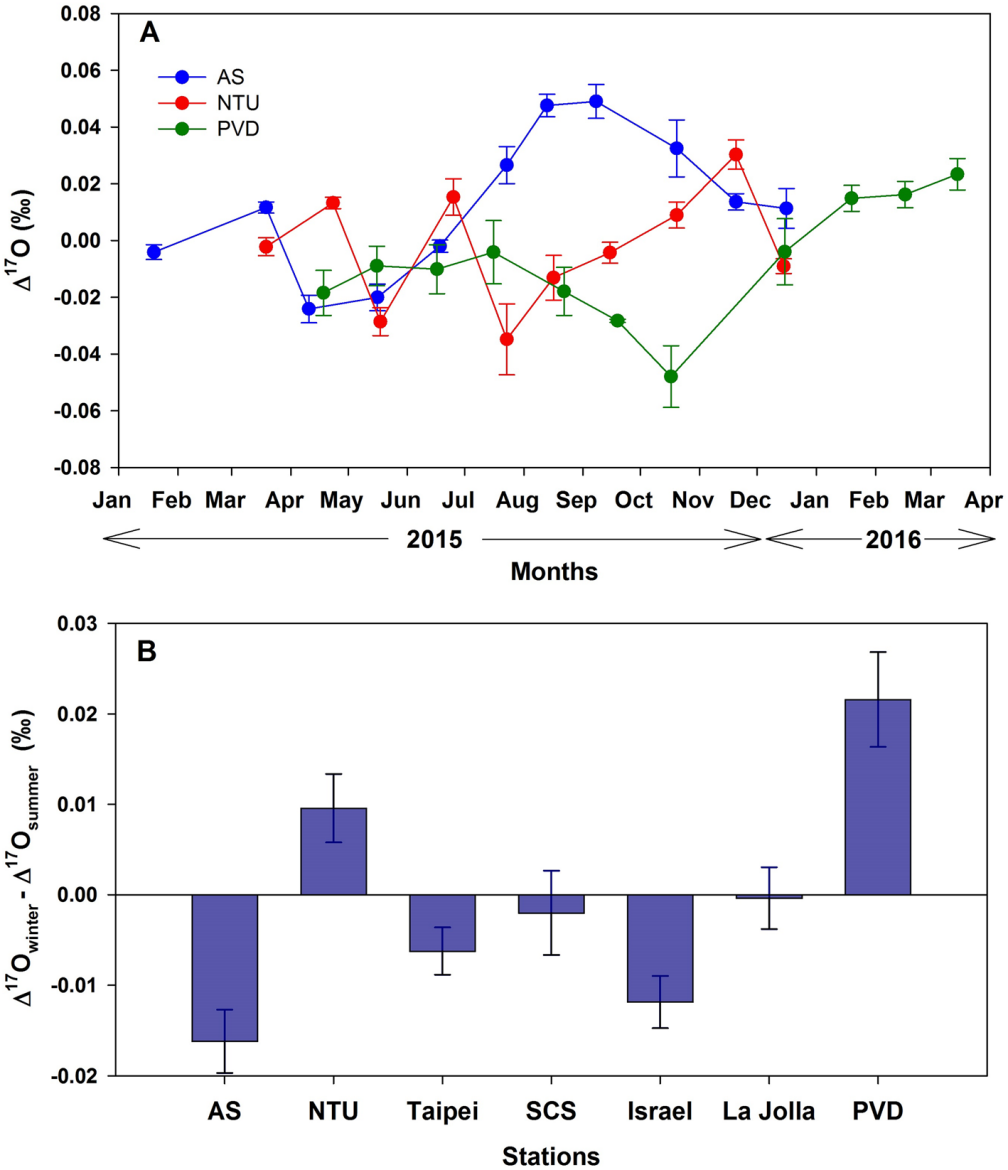
(**A**) Time series of the monthly averaged Δ^17^O values of CO_2_ from the three selected stations, where there are regular measurements in years 2015–2016. (**B**) The difference between winter (average of January–March and October–December) and summer (April–September) averaged CO_2_ Δ^17^O values. The error bars represent 1 standard error of the average.

## Results and discussion

We analyze data (see Supplementary Information for details) of the triple-oxygen isotopic compositions of surface air CO_2_ (Fig. 1 and Table 1) from six northern hemisphere sites (South China Sea and Taipei, Taiwan; Jerusalem, Israel; La Jolla, California, United States; Palos Verdes, California, United States). We derive the oxygen isotopic recycling time (τ) of CO_2_ in the atmosphere and tGPP from the integrated data set and discuss how inter/intra-hemispheric transport affects these quantities. Given that the tropospheric mixing time within each hemisphere is much shorter than the interhemispheric mixing time^45,46^ and the latter is also shorter than the CO_2_ residence time derived here (see below), the compiled data should be a valid approximation for the global average. Table 2 summarizes the model results calculated using Eqs. (2) and (3), with the errors obtained following the standard error propagation. For the current mass loading of atmospheric CO_2_ (*M*) of 828 ± 10 PgC^1,47^, the globally averaged τ given by *M*/F_sur_ (where the surface flux F_sur_ is the sum of terrestrial and oceanic gross fluxes; the former is 465 ± 60 PgC/year and the latter is 90 ± 6 PgC/year^17^) is 1.5 ± 0.2 years, assuming that the Δ^17^O value in tropospheric CO_2_ in the southern hemisphere (Δ^17^O_S_) is the same as that reported in the northern hemisphere (Δ^17^O_N_). Our sensitivity calculation finds ∂τ/∂(Δ^17^O_S_ – Δ^17^O_N_) to be 6.4 years/‰. See Supplementary Information for discussion of the evenness of intra- and inter-hemispheric Δ^17^O values. At Δ^17^O_S_ = Δ^17^O_N_, the northern hemispheric recycling time τ_N_ is 1.2 year and the southern hemispheric τ_S_ is 1.8 year. At a maximum interhemispheric difference of 0.025‰ (obtained by assuming absence of inter-hemispheric mixing; see Supplementary Information), the value of τ increases to 1.6 ± 0.2 years, with τ_N_ = 1.4 and τ_S_ = 2.0 years, consistent with those values (0.4–0.8 year and > 2 years, respectively) estimated earlier^15^; this level of interhemispheric difference was reported earlier from a global model simulation (~ 0.02‰)^20^.

**Table 2:** Estimated GPP (PgC/year) and recycling time τ (year) for this work and the literature.

	Present work	Liang et al.^17^	Welp et al.^15^	Beer et al.^13^	Hoffmann et al.^33^
Methodology	Triple oxygen (Δ^17^O–O_2_, Δ^17^O–CO_2_)	Triple oxygen (Δ^17^O–CO_2_)	ENSO single delta (δ^18^O–CO_2_)	Eddy covariance (CO_2_)	Dole effect (δ^18^O–O_2_)
Recycling time					
τ	1.5 ± 0.2	1.9 ± 0.3	0.9–1.7	N/A	N/A
τ_N_	1.2 ± 0.2	N/A	0.4–0.8	N/A	N/A
τ_S_	1.8 ± 0.2	N/A	2.6–5.0	N/A	N/A
Gross primary production					
tGPP	170–200	120 ± 30	150–170	123 ± 8	200
oGPP	90–120	N/A	N/A	N/A	91
GPP	290 ± 30	N/A	N/A	N/A	292 ± 20

With the derived terrestrial flux from Eq. (2), we can estimate the value of tGPP following Liang et al.^17^. Our best estimate for tGPP is ~ 170–200 PgC/year. The global GPP is evaluated using Eq. (3), with the values of 0.149‰ and 0.202‰ (referenced to tropospheric O_2_) for terrestrial and oceanic photosynthetic O_2_, respectively. Following the photosynthetic O_2_ scenario of Luz and Barkan^25^, we derived a global GPP of 290 ± 30 PgC/year at tGPP of ~ 170–200 PgC/year obtained above from CO_2_, in excellent agreement with the most recent value (292 ± 20 PgC/year) from the atmospheric O_2_ Dole effect^33^. The derived global GPP from O_2_ is insensitive to the partitioning between the terrestrial and oceanic components. For example, assuming an equal flux of tGPP and oGPP, global GPP changes to 283 ± 30 PgC/year, within error of the value estimated above (see Supplementary Information). The global carbon budget obtained from this work is summarized in Fig. 2 and Table 2.

The interplay of climate and biogeochemical cycles is yet to be fully understood^1,8^. Several lines of evidence have shown that the global carbon cycle has changed noticeably^4,5,48^. For example, an unexpected reversal of C_3_ versus C_4_ grass response to elevated CO_2_ noted recently from a 20-year field experiment posed a great challenge to the community and modelers that the current knowledge of carbon cycles remains insufficient in assessing the changing ecosystem^7^. Despite the progress made in attempting to model the carbon cycles, caveats remain^1,6,8,15^. Central components that need more study include gross fluxes of CO_2_ between reservoirs such as terrestrial and oceanic gross primary productivities. This work provides a lengthy data set from a new perspective with wide geographical coverage and resolution. The triple-oxygen isotopic composition of CO_2_ constrains the global oxygen isotopic residence time of CO_2_ in the atmosphere to 1.5 ± 0.2 years, compared to 0.9–1.7 years^15,17^ or longer^10,11^. The terrestrial gross flux is quantified to be 550 ± 60 PgC/year, falling in the range reported in the literature, 200–660 PgC/year^10,11,15^. Our best estimate of tGPP is ~ 170–200 PgC/year, compared to the current models of tGPP of ~ 110–150 PgC/year^8^, suggesting that the models should be revisited to achieve a full understanding of ecosystem changes due to the changing climate and environmental factors^4–7,49^. The inferred oGPP is ~ 90–120 PgC/year, verifying those reported previously^32,33^ but from an independent perspective. Because of the isotope recycling time of CO_2_, the spatial inhomogeneity of Δ^17^O obtained between localities shows that the commonly used δ values can be applied to Δ^17^O to refine knowledge of the flux partitioned between respiration/soil invasion, photosynthesis, and air-sea exchange.

In short, with constraints from the triple oxygen isotopic compositions in atmospheric CO_2_ and O_2_, we robustly derive the terrestrial and oceanic gross fluxes of oxygen on the global scale, done by averaging the CO_2_ data (because of its lifetime in the atmosphere, O_2_ is well-mixed) over the various localities and time. We note that the El Nino-modulated changes in the global carbon cycle reported by Thiemens et al.^16^ are, however, not seen in the new dataset during the 2014–2016 event (the strength of this El Nino event was slightly weaker than the 1997–1998 one), inferring a hitherto unidentified response in the global carbon cycle to climatic effects. Indeed, from a recent analysis of CO_2_ concentrations in western Pacific regions^50^, the amplitude of inter-annual climatic modulations of ENSO and Pacific Decadal Oscillation-like variabilities is ~ 5 ppm in the lower troposphere and reduces to ~ 0.5 ppm in the mid-troposphere. How this is translated into gross fluxes, reflected in the Δ^17^O of CO_2_, is yet to be quantified. However, further analysis of the data presented in Fig. 1 shows significant and systematic spatial and temporal variations of Δ^17^O in CO_2_ (Fig. 4). The maximum seasonal changes are found to be similar to the reduction of Δ^17^O reported earlier during the 1997–1998 El Nino period^16^, though no apparent seasonal variation in Δ^17^O was seen during 1997–1998^16^. Comparing AS and PVD, the values during the second half of 2015 (July–November) are drastically different, being enhanced by as much as ~ 0.04‰ for the former and depleted by ~ 0.05‰ for the latter. The features and magnitudes are inconsistent with a current global model simulation^20^ where, for both locations, the model predicted the Δ^17^O values would be higher by ~ 0.02‰ during March–August than during January–February and September–December. More astonishingly, AS and NTU do not vary coherently, despite their close proximity. Overall, AS and Israel each show a seasonal maximum in summer, in contrast to the winter high at PVD. The analysis suggests that the CO_2_ recycling time in the northern hemisphere is not much longer than one year because of the rather short hemispheric mixing time of less than ~ 4 months^46^, verifying the result of ~ 1 year recycling time derived above. However, we defer detailed analysis of the inter-annual and intra-annual variations to a later study, when longer data sets are available. Finally, we note that the Δ^17^O approach, with proper model assimilation^20^, can be used in the future to quantify and refine the gross fluxes, which were not available, including on local and regional scales^51^.

## Supplementary Information

Supplementary Information.

Supplementary Table S3.

## Data Availability

All data generated or analyzed during this study are either included in this paper [and its Supplementary Information files] or available in other published articles referred in Table 1.

## References

[1] IPCC (2014). Climate Change 2013: The Physical Science Basis: Contribution of Working Group I to the Fifth Assessment Report of IPCC the Intergovernmental Panel on Climate Change.

[2] Le Quéré C (2018). Global carbon budget 2018. Earth Syst. Sci. Data.

[3] IPCC (2021). Climate Change 2021: The Physical Science Basis: Contribution of Working Group I to the Sixth Assessment Report of the Intergovernmental Panel on Climate Change.

[4] Graven H (2013). Enhanced seasonal exchange of CO_2_ by northern ecosystems since 1960. Science.

[5] Campbell J (2017). Large historical growth in global terrestrial gross primary production. Nature.

[6] Winkler AJ, Myneni RB, Alexandrov GA, Brovkin V (2019). Earth system models underestimate carbon fixation by plants in the high latitudes. Nat. Commun..

[7] Reich PB, Hobbie SE, Lee TD, Pastore MA (2018). Unexpected reversal of C_3_ versus C_4_ grass response to elevated CO_2_ during a 20-year field experiment. Science.

[8] Piao S (2013). Evaluation of terrestrial carbon cycle models for their response to climate variability and to CO_2_ trends. Glob. Change Biol..

[9] Lawrence DM (2011). Parameterization improvements and functional and structural advances in version 4 of the Community Land Model. J. Adv. Model. Earth Syst..

[10] Ciais P (1997). A three-dimensional synthesis study of δ^18^O in atmospheric CO_2_: 1. Surface fluxes. J. Geophys. Res. Atmos..

[11] Farquhar GD (1993). Vegetation effects on the isotope composition of oxygen in atmospheric CO_2_. Nature.

[12] Field CB, Behrenfeld MJ, Randerson JT, Falkowski P (1998). Primary production of the biosphere: Integrating terrestrial and oceanic components. Science.

[13] Beer C (2010). Terrestrial gross carbon dioxide uptake: Global distribution and covariation with climate. Science.

[14] Wingate L (2009). The impact of soil microorganisms on the global budget of δ^18^O in atmospheric CO_2_. Proc. Natl. Acad. Sci..

[15] Welp LR (2011). Interannual variability in the oxygen isotopes of atmospheric CO_2_ driven by El Niño. Nature.

[16] Thiemens MH, Chakraborty S, Jackson TL (2014). Decadal Δ^17^O record of tropospheric CO_2_: Verification of a stratospheric component in the troposphere. J. Geophys. Res. Atmos..

[17] Liang M-C, Mahata S, Laskar AH, Thiemens MH, Newman S (2017). Oxygen isotope anomaly in tropospheric CO_2_ and implications for CO_2_ residence time in the atmosphere and gross primary productivity. Sci. Rep..

[18] Laskar AH, Mahata S, Bhattacharya SK, Liang MC (2019). Triple oxygen and clumped isotope compositions of CO_2_ in the middle troposphere. Earth Space Sci..

[19] Cuntz M (2003). A comprehensive global three-dimensional model of δ^18^O in atmospheric CO_2_: Mapping the atmospheric signal. J. Geophys. Res. Atmos..

[20] Koren G (2018). Global 3D Simulations of the Triple Oxygen Isotope Signature Δ^17^O in Atmospheric CO_2_. J. Geophys. Res. Atmos..

[21] Francey RJ, Tans PP (1987). Latitudinal variation in oxygen-18 of atmospheric CO_2_. Nature.

[22] Gillon J, Yakir D (2001). Influence of carbonic anhydrase activity in terrestrial vegetation on the ^18^O content of atmospheric CO_2_. Science.

[23] Yoshimura K, Kanamitsu M, Noone D, Oki T (2008). Historical isotope simulation using reanalysis atmospheric data. J. Geophys. Res. Atmos..

[24] Prokopenko MG, Pauluis OM, Granger J, Yeung LY (2011). Exact evaluation of gross photosynthetic production from the oxygen triple-isotope composition of O_2_: Implications for the net-to-gross primary production ratios. Geophys. Res. Lett..

[25] Luz B, Barkan E (2011). Proper estimation of marine gross O_2_ production with ^17^O/^16^O and ^18^O/^16^O ratios of dissolved O_2_. Geophys. Res. Lett..

[26] Luz B, Barkan E, Bender ML, Thiemens MH, Boering KA (1999). Triple-isotope composition of atmospheric oxygen as a tracer of biosphere productivity. Nature.

[27] Hoag K, Still C, Fung I, Boering K (2005). Triple oxygen isotope composition of tropospheric carbon dioxide as a tracer of terrestrial gross carbon fluxes. Geophys. Res. Lett..

[28] Hofmann M (2017). Atmospheric measurements of Δ^17^O in CO_2_ in Göttingen, Germany reveal a seasonal cycle driven by biospheric uptake. Geochim. Cosmochim. Acta.

[29] Thiemens MH (2006). History and applications of mass-independent isotope effects. Annu. Rev. Earth Planet. Sci..

[30] Hofmann ME, Horváth B, Pack A (2012). Triple oxygen isotope equilibrium fractionation between carbon dioxide and water. Earth Planet. Sci. Lett..

[31] Barkan E, Luz B (2012). High-precision measurements of ^17^O/^16^O and ^18^O/^16^O ratios in CO_2_. Rapid Commun. Mass Spectrom..

[32] Bender M, Sowers T, Labeyrie L (1994). The Dole effect and its variations during the last 130,000 years as measured in the Vostok ice core. Glob. Biogeochem. Cycles.

[33] Hoffmann G (2004). A model of the Earth's Dole effect. Glob. Biogeochem. Cycles.

[34] Uemura R, Barkan E, Abe O, Luz B (2010). Triple isotope composition of oxygen in atmospheric water vapor. Geophys. Res. Lett..

[35] Landais A, Barkan E, Yakir D, Luz B (2006). The triple isotopic composition of oxygen in leaf water. Geochim. Cosmochim. Acta.

[36] Dai A (2006). Recent climatology, variability, and trends in global surface humidity. J. Clim..

[37] Young ED, Yeung LY, Kohl IE (2014). On the Δ^17^O budget of atmospheric O_2_. Geochim. Cosmochim. Acta.

[38] Brenninkmeijer C, Röckmann T (1996). Russian doll type cryogenic traps: Improved design and isotope separation effects. Anal. Chem..

[39] Barkan E, Musan I, Luz B (2015). High-precision measurements of δ^17^O and ^17^O_excess_ of NBS19 and NBS18. Rapid Commun. Mass Spectrom..

[40] Mahata S, Bhattacharya S, Wang C-H, Liang M-C (2013). Oxygen isotope exchange between O_2_ and CO_2_ over hot platinum: An innovative technique for measuring Δ^17^O in CO_2_. Anal. Chem..

[41] Laskar AH, Liang M-C (2016). Clumped isotopes in near-surface atmospheric CO_2_ over land, coast and ocean in Taiwan and its vicinity. Biogeosciences.

[42] Laskar AH, Mahata S, Liang M-C (2016). Identification of anthropogenic CO_2_ using triple oxygen and clumped isotopes. Environ. Sci. Technol..

[43] Mahata S, Bhattacharya S, Liang MC (2016). An improved method of high-precision determination of Δ^17^O of CO_2_ by catalyzed exchange with O_2_ using hot platinum. Rapid Commun. Mass Spectrom..

[44] Thiemens MH, Jackson T, Zipf EC, Erdman PW, van Egmond C (1995). Carbon dioxide and oxygen isotope anomalies in the mesosphere and stratosphere. Science.

[45] Jacob DJ, Prather MJ, Wofsy SC, McElroy MB (1987). Atmospheric distribution of ^85^Kr simulated with a general circulation model. J. Geophys. Res. Atmos..

[46] Lal D (1966). Characteristics of global tropospheric mixing based on man-made C^14^, H^3^, and Sr^90^. J. Geophys. Res..

[47] Joos F (2013). Carbon dioxide and climate impulse response functions for the computation of greenhouse gas metrics: a multi-model analysis. Atmos. Chem. Phys..

[48] Lobell DB, Schlenker W, Costa-Roberts J (2011). Climate trends and global crop production since 1980. Science.

[49] Lobell DB, Gourdji SM (2012). The influence of climate change on global crop productivity. Plant Physiol..

[50] Hsueh Y-H (2021). East Asian CO_2_ level change caused by Pacific decadal oscillation. Remote Sens. Environ..

[51] Ryu Y, Berry JA, Baldocchi DD (2019). What is global photosynthesis? History, uncertainties and opportunities. Remote Sens. Environ..

[52] Luz B, Barkan E (2010). Variations of ^17^O/^16^O and ^18^O/^16^O in meteoric waters. Geochim. Cosmochim. Acta.

