# Supplementary material for: New constraints of terrestrial and oceanic global gross primary productions from the triple oxygen isotopic composition of atmospheric CO2 and O2
Source: Sci Rep. 2023 Feb 7;13:2162. doi: 10.1038/s41598-023-29389-z (PMC9905602; doi:10.1038/s41598-023-29389-z)
Supplement: Supplementary file 1 — Supplementary Information. [file 41598_2023_29389_MOESM1_ESM.docx]

Supplementary Information

New constraints of terrestrial and oceanic global gross primary productions from the triple oxygen isotopic composition of atmospheric CO_2_ and O_2_

Mao-Chang Liang^1*^, Amzad H. Laskar^2^, Eugeni Barkan^3^, Sally Newman^4^_,_ Mark H. Thiemens^5*^_,_ Ravi Rangarajan^1,6^

^1^Institute of Earth Sciences, Academia Sinica, Taipei, Taiwan

^2^Physical Research Laboratory, Ahmedabad, Gujarat, India

^3^Institute of Earth Sciences, Hebrew University of Jerusalem, Jerusalem, Israel

^4^Division of Geological and Planetary Science, California Institute of Technology, Pasadena, California, USA; now at Bay Area Air Quality Management District, San Francisco, USA

^5^Department of Chemistry and Biochemistry, University of California at San Diego, La Jolla, California, USA

^6^Now at Department of Public Health, College of Health Sciences, University of Doha for Science and Technology, Doha, Qatar

*To whom correspondence should be addressed: mcl@gate.sinica.edu.tw, mthiemens@ucsd.edu

**Leaf evapotranspiration for the triple-oxygen isotope variation in tropospheric CO_2_**

Exchanging oxygen isotopes with water is the major process in determining CO_2_ fluxes from the biosphere/hydrosphere. The associated λ is well defined experimentally^1,2^ and the fluxes (e.g., the terrestrial flux - the cycling flux between the terrestrial biosphere and atmosphere, inferred from the oxygen isotopic composition of CO_2_) can then be constrained (cf.^3^). Given that the carbon flux estimation presented in this paper is based on the deviations of the oxygen anomalies of reservoirs/processes from those measured in atmospheric CO_2_, it is natural to take the λ value best describing the variation of the triple-oxygen isotopic partitioning in tropospheric CO_2_. Processes that affect CO_2_ isotopocules in the troposphere are terrestrial, oceanic, and anthropogenic, with the first being dominant (e.g., see Figure 4B of Liang et al. ^4^ for their relative importance). In the terrestrial biosphere, leaf water largely governs the oxygen isotope composition of CO_2_ (ref.^5-11^). The leaf water isotopic composition is known to be affected by evaporation at reduced relative humidity. It has been found previously that the evapotranspiration λ value in water oxygen isotopic systematics is a strong function of ambient air relative humidity^12^, whereas dependence on other meteorological variables such as temperature and soil water isotopic composition has not been observed. We set λ = 0.516, as it represents the evapotranspiration λ at 75% relative humidity, a globally averaged humidity near the surface^13^. The regions where vegetation is dense generally have relative humidity 75±5% (ref.^13^), corresponding to λ = 0.5160±0.0004 (with all possible errors included; re-calculated from Landais et al. ^12^, following the linear definition of λ defined in the main text). As a result, we take 0.516 as our reference value.

Indeed, from the CO_2_ data acquired in the western Pacific (with a total of 327 good measurements, after excluding one outlier having an extremely low Δ^17^O value of -0.025 ‰), the linear regression yields a slope of 0.516±0.002 in the δ^17^O versus δ^18^O plot, conforming with the average relative humidity of 76±4% in the region. Sensitivity tests show that a shift of 0.001 in λ does not impact the finally derived tGPP much; a change of +0.001 in λ at ~0.516 yields a change of +8 PgC/yr in tGPP, or ~24 PgC/yr in the terrestrial gross flux, F_t_ which reduces the recycling time τ by 0.06 year.

**Sensitivity of the terrestrial and oceanic processes in the CO_2_ cycling**

The sensitivity of the terrestrial and oceanic processes can be assessed following equation (2) of the main text. That is,

$\sum_{i} F_{i}\times(\Delta^{17}O_{i}-\Delta^{17}O)=0$ (S3)

where “i” is the individual process being evaluated. In general, the greater the abs(Δ^17^O_i_ - Δ^17^O) value, the higher the sensitivity of the derived F_i_ to the process “i.” As a result, the anthropogenic is the most sensitive process and oceanic the least, a consequence of mass balance of the Δ^17^O isoflux. Overall, terrestrial processes are roughly equally sensitive. For example, with a reduction of 100 PgC/yr in soil invasion, to maintain the same level of the isoflux from the terrestrial biosphere, one has to increase either respiration by 100 PgC/yr, or leaf retroflux by 74 PgC/yr. For oceanic, the increase is 333 PgC/yr, in order to balance the reduction of isoflux from the terrestrial biosphere. For anthropogenic CO_2_, the increase is 21 PgC/yr only. The above exercise clearly demonstrates the sensitivity of the triple-oxygen isotope approach to the overall terrestrial processes, but the total budget is less sensitive to a particular biological process in the terrestrial biosphere due to the common source of water, meteoric water. A bar chart of the sensitivity is shown in Figure 3 of the main text.

**Evenness of the intra- and inter-hemispheric Δ^17^O in CO_2_**

Here, we estimate the spatial homogeneity of annually averaged Δ^17^O values and discuss how intra- and inter-hemispheric transport affects the terrestrial gross flux F_t_ and tGPP. The tropospheric mixing time in each hemisphere ranges from 2-3 weeks to ~4 months^14^. Largely affected by Hadley circulation, it is known that the exchange of air masses across ~30° latitude is inefficient and the efficiency of the mixing is seasonally dependent. As a result, the exchange time varies between ~0.3 and 4 months, with an average of ~3 months^14^; see also Figure S1 for the horizontal wind of the northern hemisphere of the order of 1 m s^-1^, implying the mixing time is about a few months. Within each latitude bin (0-30° and 30-90°), the mixing time is in general less than 3 weeks, in either hemisphere. Inter-hemispheric mixing time is about 1.1 years^14,15^. With these time scales, along with the oxygen isotope turnover time, the spatial homogeneity of Δ^17^O in CO_2_ can be estimated, even before an extensive set of global data comes available.

To start, we adopt the carbon cycling model results from the National Center for Atmospheric Research Community Climate System Model coupled with the Community Land Model 4.0 (CLM4) for 20^th^ century runs^16^; there are 30 ensemble runs. The main reason for this model to be selected for comparison is that the model generates tGPP of 147 PgC/yr, one of highest values among the IPCC AR5 models^16,17^, closer to the new estimate derived in this work and reported by Welp et al. ^3^. The model can also produce a reasonable global response of the terrestrial biosphere to the changing climate reflected in temperature, precipitation, and atmospheric CO_2_ level (see Piao et al. ^18^ for details).

We first estimate the difference in Δ^17^O in CO_2_ between the hemispheres. The respective Δ^17^O budget, expanded from equation (S2) for each hemisphere (N denotes the northern hemisphere and S the southern hemisphere), following Hoag et al. ^19^, can be written as follows.

$\sum_{i} F_{N,i}\times\left( \Delta^{17}O_{i}-\Delta^{17}O_{N} \right)+\frac{M_{S}}{\tau_{h}}\times(\Delta^{17}O_{S}-\Delta^{17}O_{N})=0$ (S1)

$\sum_{i} F_{S,i}\times\left( \Delta^{17}O_{i}-\Delta^{17}O_{S} \right)+\frac{M_{N}}{\tau_{h}}\times(\Delta^{17}O_{N}-\Delta^{17}O_{S})=0$, (S2)

where M_N_ (give value) and M_S_ (give value) are the CO_2_ mass loading of each hemisphere and τ_h_ the inter-hemispheric mixing time of 1.1 year. Using the tGPP obtained by CLM4 (northern tGPP is 84 PgC/yr and southern tGPP is 63 PgC/yr), the hemispheric Δ^17^O difference is 0.025 ‰, assuming absence of inter-hemispheric transport. Including the 1.1 year inter-hemispheric mixing time, the difference reduces to 0.006 ‰ (=0.025 ‰ × e^-(1.5 year)/(1.1 year)^, at 1.5 year global oxygen isotope recycling time, τ). This difference would increase our best estimated τ by 0.04 year, much less than the error of 0.2 year reported in the main text. Here, we have assumed that the cross-tropopause exchange is the same between the hemispheres e.g., see Škerlak et al. ^20^.

We next estimate how intra-hemispheric transport and mixing affects the Δ^17^O values of atmospheric CO_2_. Given that the mixing time (<3 weeks) in each latitude bin is much less than the derived oxygen isotope residence time (1.2 years, derived in this work versus as short as 0.4 year reported by Welp et al. ^3^ in the northern hemisphere), the Δ^17^O inhomogeneity is less than e^-(0.4 years)/(3 weeks)^ = 0.1 %. The largest contrast comes from the cross 30° latitude transport in the northern hemisphere. If the most conservative value of the oxygen isotope residence time of 0.4 year from Welp et al. ^3^ is taken, the percentage difference of Δ^17^O between 0-30° and 30-90° is e^-(0.4 year)/(3 months)^ = 37 %. We then take the tGPP and soil respiration values from the CLM4 model and assume soil invasion is the same as soil respiration (the most active soil invasion scenario reported by Wingate et al. ^9^). The estimated Δ^17^O difference is about 0.1 ‰, or (0.1 ‰ ×37 %) = 0.037 ‰ after considering cross 30° latitude transport; the value at the low latitude band is lower than that at the high latitude band (a consequence of upwelling air transport at low latitudes due to Hadley cell circulation and downwelling transport of air masses from the stratosphere at high latitudes; e.g., see Holton et al. ^21^ and Škerlak et al. ^20^ for details). Such difference, however, has not been found. Instead, high latitude data from Hofmann et al. ^22^ show a lower value (by ~0.05 ‰) compared to lower latitude data; the data of Hofmann et al. ^22^ may be subject to some unknown systematic biases, and as a result, that conclusion of the high latitude depletion needs to be further verified. However, careful examination shows that Taiwan and SCS are likely in the low latitude band (0-30°), whereas Israel and USA are in the high latitude band. This separation is supported by the cross-tropopause exchange pattern analyzed and obtained by Škerlak et al. ^20^. Within error, no statistically meaningful difference between the two bands is observed (see Table 1 of the main text). If an error of 0.01 ‰ is assumed for the difference, the inferred oxygen isotope turnover time is 0.9 year in the northern hemisphere, consistent with the time of 1.2 year reported in the main text.

In short, our model predicts a negligible difference (<0.01 ‰) in annually averaged Δ^17^O between latitudes and between hemispheres, the same conclusion reported earlier by Hoag et al. ^19^.

**Terrestrial gross primary productivity, tGPP**

Plant uptake scenarios affect the estimates of GPP and soil invasion. GPP can be estimated as follows:

$0.88\times GPP = \frac{F_{t}- F_{s}}{\theta_{eq}\kappa_{c}+1}$, (S4)

where F_s_ is soil invasion, *θ_eq_* represents the degree of hydration of CO_2_ inside stomata, and *κ_c_* is a measure of stomatal conductance which can be expressed by

*κ_c_* = *C_c_*/(*C_a_* – *C_c_*), (S5)

where *C_c_* is the CO_2_ concentration in chloroplasts at the site of CO_2_ hydration and *C_a_* is the atmospheric concentration. The factor 0.88 is used to account for leaf respiration^23^. For C_3_ plants, *C_c_/C_a_* = 2/3; for C_4_ plants, *C_c_/C_a_* = 1/3, assuming that *C_c_* is equal to intracellular CO_2_ concentration^24^. A globally averaged *C_c_/C_a_* is 0.57 or *κ_c_* = 1.33 (ref.^3,11^). *θ_eq_*, however, is variable^11,24^ and a currently globally averaged value is 0.78 (ref.^11^). Sensitivity analysis shows that over the range of *κ_c_* bound between C_3_ and C_4_ plants, the change in tGPP is -4 PgC/yr for every 0.1 increase in *κ_c_*; the calculation is done at *θ_eq_* of 0.78. The sensitivity is -8 PgC/yr per 0.1 increase in *θ_eq_*; the calculation is performed at *κ_c_* = 1.33. It was pointed out previously^24^ that the current estimate of *θ_eq_*, reported by Gillon and Yakir ^11^ is likely overestimated, but we think the overestimation is not too much, given the extent of global C_4_ plants compared to C_3_ plants. A likely range of globally averaged *θ_eq_* is ~0.6-0.7, which corresponds to the value of tGPP of ~175-185 PgC/yr.

Another factor mentioned above is the selection of λ. At λ ≈ 0.516, the sensitivity to the tGPP is +8 PgC/yr per +0.001 change. The dependence is not linear; the higher the value of λ, the larger the sensitivity of tGPP (and τ) to λ. At λ ≈ 0.520-0.523, the sensitivity increases to +16 PgC/yr, doubling the sensitivity of that at λ ≈ 0.516. The higher sensitivity is because of the equilibration slope of water-CO_2_ in the dual-oxygen isotope space, which is 0.5229. At λ of 0.518, *κ_c_* of 1.33, and *θ_eq_* of 0.7, the tGPP value is 200 PgC/yr.

Overall, based on the assessments presented above, a choice of λ value between 0.516 and 0.518 for describing the Δ^17^O budget is reasonable, and *θ_eq_* value around 0.7 is preferred. The selection yields a best estimate of tGPP of ~170-200 PgC/yr.

**Global primary productivity**

Utilization of the triple-oxygen isotopic composition of molecular oxygen in the atmosphere for GPP has been established ^25^. Unlike CO_2_, O_2_ does not exchange isotopes with water in a traditional way. During photosynthetic biomass production, O_2_ preserves its isotopic composition before getting modified by respiration, following a well-defined slope in the triple-oxygen isotope plot. Following the exact analytical formula for GPP, the globally averaged O_2_ GPP at steady state can be written as follows:

GPP×(δ^17^O_pe_ – 0.516×δ^18^O_pe_) + F_st_×(δ^17^O_st_ – 0.516×δ^18^O_st_) = 0, or (S6)

GPP×Δ^17^O_pe_ + F_st_×Δ^17^O = 0, (S7)

where δ^17^O_pe_ and δ^18^O_pe_ are the effective photosynthetic O_2_ delta values from the biosphere and δ^17^O_st_ and δ^18^O_st_ are the values for O_2_ from the stratosphere; all the delta values are referenced with respect to tropospheric O_2_. The coefficient 0.516 is taken from the recommendation of Luz and Barkan ^26^. The value of F_st_×(δ^17^O_st_ – 0.516×δ^18^O_st_) is taken from Liang et al. ^27^ derived for the CO_2_ Δ^17^O isoflux across the tropopause. Assuming steady state in CO_2_-O_3_-O_2_ coupled chemistry, the positive Δ^17^O isoflux in CO_2_ is balanced by the negative Δ^17^O isoflux in O_2_. The latter is then balanced by a positive isoflux from the biosphere.

GPP can be written as functions of tGPP and oGPP, i.e.,

GPP×Δ^17^O_pe_ = tGPP×Δ^17^O_p,t_ + oGPP×Δ^17^O_p,o_ (S8)

The photosynthetic values of δ^17^O_p_ and δ^18^O_p_ (in the VSMOW substrate) taken from Luz and Barkan ^26^ are -10.126 ‰ and -20.014 ‰, respectively, with respect to atmospheric O_2_; the Δ^17^O_p_ value is 0.201 ‰. The actual values include substrate water Δ^17^O. That is,

Δ^17^O_p,t_ = Δ^17^O_p_ + Δ^17^O_mw_ = 0.149 ‰

Δ^17^O_p,o_ = Δ^17^O_p_ + Δ^17^O_o_ = 0.202 ‰

The final GPP depends on the partition of tGPP and oGPP. Assuming equally partitioned^28^, GPP is 283±30 PgC/yr (the error, calculated following the standard error propagation in equation (S8), is mainly from the measurement uncertainty in photosynthetic O_2_; see Table 1 of the main text for the errors). If we take the derived tGPP from the main text, the GPP increases to 293 PgC/yr. If Δ^17^O_p_ is the same as the substrate water value, the GPP increases further to 335 PgC/yr. We think it is more reasonable to use the same photosynthetic O_2_ delta values as the marine ones^26^ for terrestrial photosynthetic O_2_. Therefore, the value of 293 PgC/yr should be taken. The sensitivity of the GPP to the tGPP/GPP ratio is insignificant. Changing the ratio of tGPP/GPP from 0.5 to 0.9 changes the GPP value weakly from 283 PgC/yr to 321 PgC/yr.

**Table S1:** VSMOW2-CO_2_ equilibration at 25 °C, determined following Liang et al. ^4^. The values of δ^17^O are calculated using the scale determined and adopted previously^4,29^.

| No. | δ^17^O (‰) | δ^18^O (‰) | ^17^α | ^18^α | ln(^17^α)/ln(^18^α) |
| --- | --- | --- | --- | --- | --- |
| 1 | 21.348 | 41.159 | 1.02135 | 1.04116 | 0.5237 |
| 2 | 21.361 | 41.159 | 1.02136 | 1.04116 | 0.5240 |
| 3 | 21.373 | 41.232 | 1.02137 | 1.04123 | 0.5234 |
| 4 | 21.384 | 41.180 | 1.02138 | 1.04118 | 0.5243 |
| 5 | 21.394 | 41.220 | 1.02139 | 1.04122 | 0.5241 |
| 6 | 21.365 | 41.180 | 1.02137 | 1.04118 | 0.5239 |
| 7 | 21.450 | 41.328 | 1.02145 | 1.04133 | 0.5241 |
| 8 | 21.455 | 41.328 | 1.02146 | 1.04133 | 0.5242 |
| 9 | 21.387 | 41.232 | 1.02139 | 1.04123 | 0.5237 |
| mean | 21.391 | 41.224 | 1.02139 | 1.04122 | 0.5239 |
| SD | 0.038 | 0.065 | 0.00004 | 0.00007 | 0.0003 |
| SE | 0.013 | 0.022 | 0.00001 | 0.00002 | 0.0001 |

**Table S2:** AS-2 CO_2_ determined in Israel, following Barkan et al. ^30^. The values of δ^17^O are determined using ^17^α of 1.021254 and ^18^α of 1.041036. ^17^Δ = ln(1+δ^17^O) – 0.516×ln(1+δ^18^O). Our Academia Sinica nominal ^17^Δ value is 0.161 ‰, obtained with calibration against a determined AS-1 reported by Liang and Mahata ^31^.

| No. | δ^17^O (‰) | δ^18^O (‰) | ^17^Δ (‰) |
| --- | --- | --- | --- |
| 1 | 18.857 | 36.61 | 0.128 |
| 2 | 18.859 | 36.61 | 0.131 |
| 3 | 18.863 | 36.61 | 0.134 |
| 4 | 18.858 | 36.61 | 0.129 |
| 5 | 18.854 | 36.61 | 0.125 |
| mean | 18.858 | 36.61 | 0.129 |
| SD | 0.003 | N/A | 0.003 |
| SE | 0.001 | N/A | 0.001 |

**Table S3:** Summary of the new CO2 data obtained in this work from South China Sea (SCS), Israel, and Palos Verdes (PVD), provided in a separate supplementary table file.

**Figure S1:** Wind vectors and wind speed (color coded, in m/s) taken from NCEP/NCAR Reanalysis^32^ for year 2015 at 700, 850, 925, and 1000 mbar. Three different time periods are averaged: annual, January-June, and July-December. NCEP Reanalysis data are provided by the NOAA/OAR/ESRL PSD, Boulder, Colorado, USA, from their Web site at http://www.esrl.noaa.gov/psd/. The images are provided by the NOAA/ESRL Physical Sciences Laboratory, Boulder Colorado from their Web site at http://psl.noaa.gov/.

**
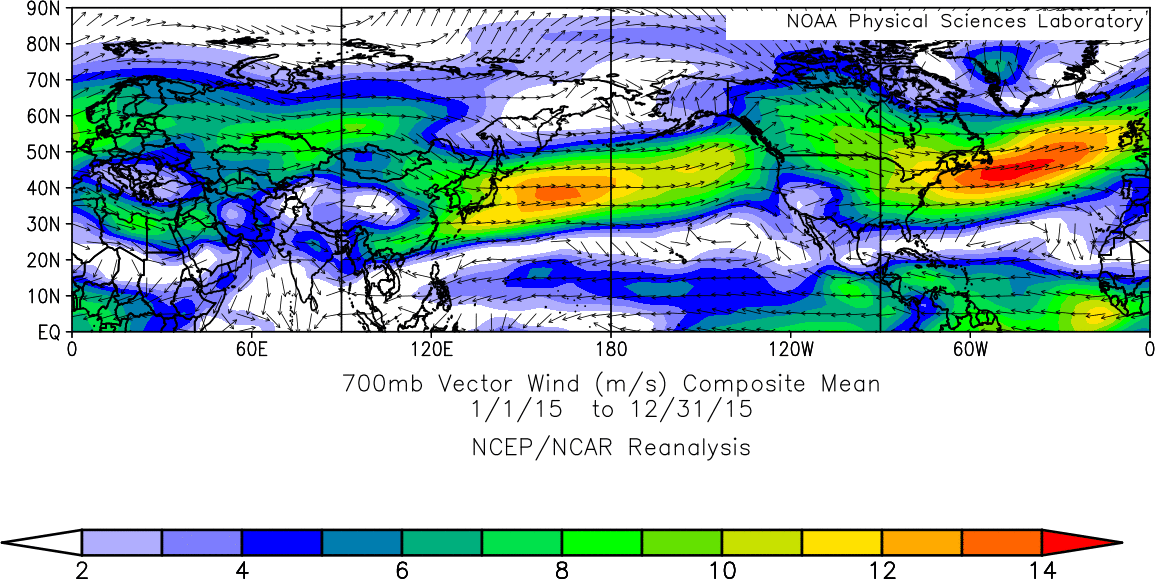

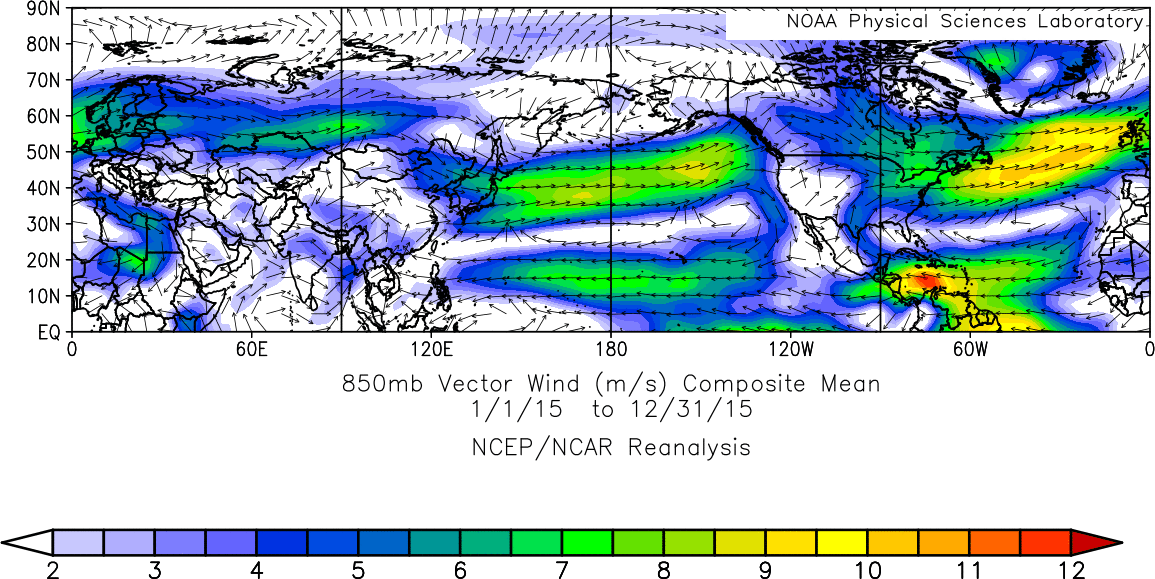

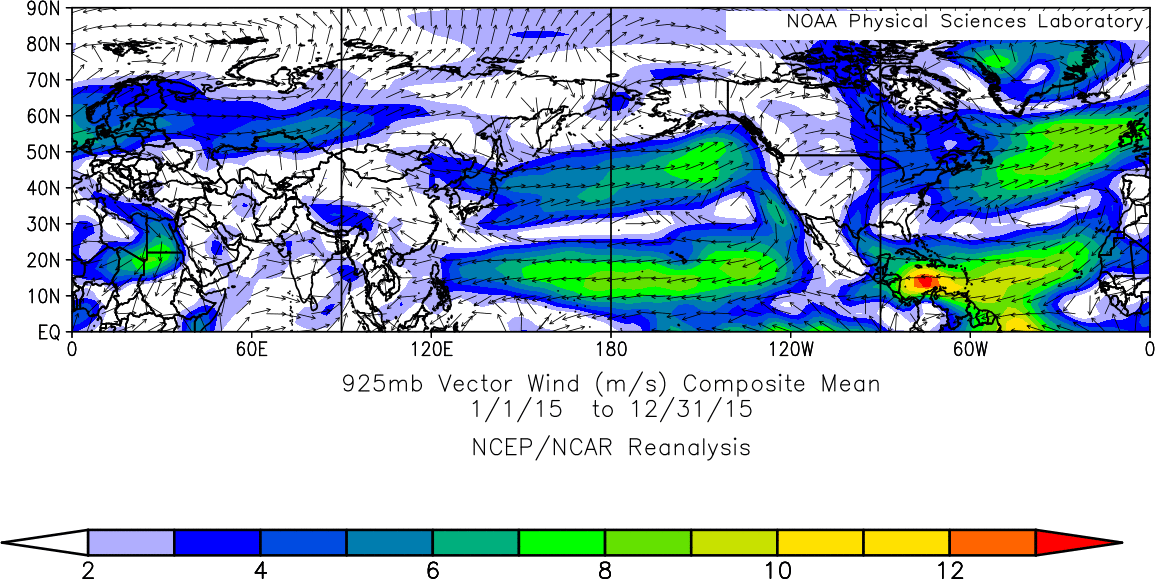

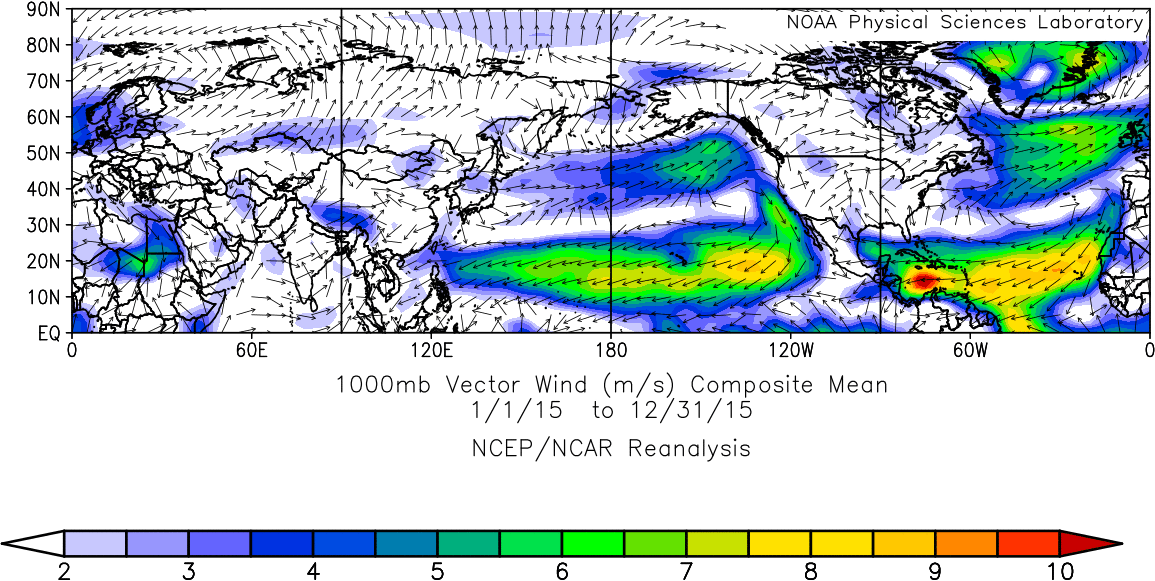
**

**
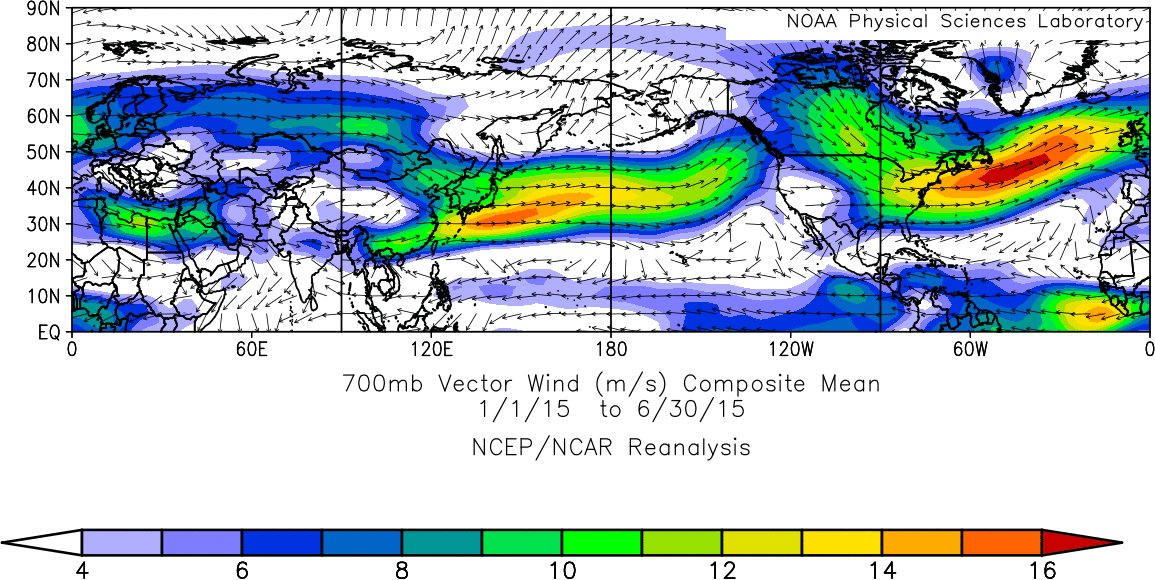

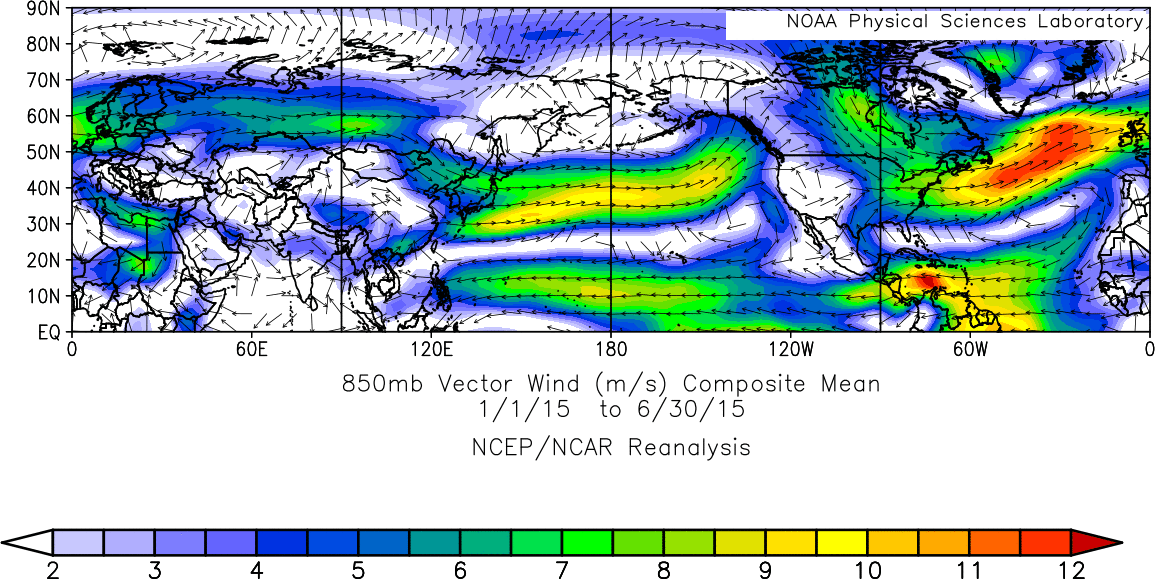

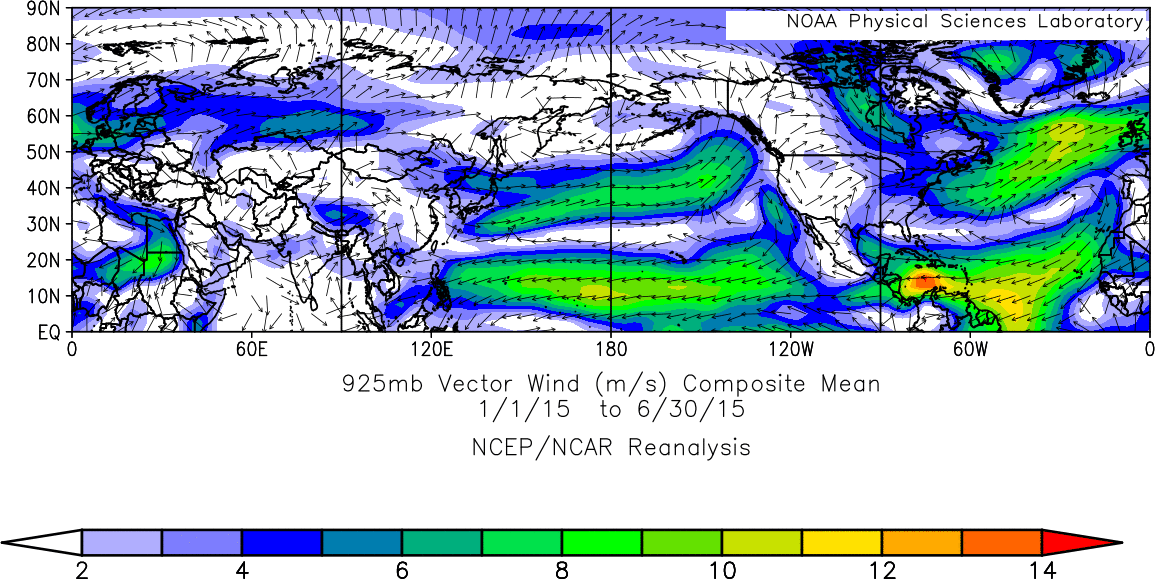

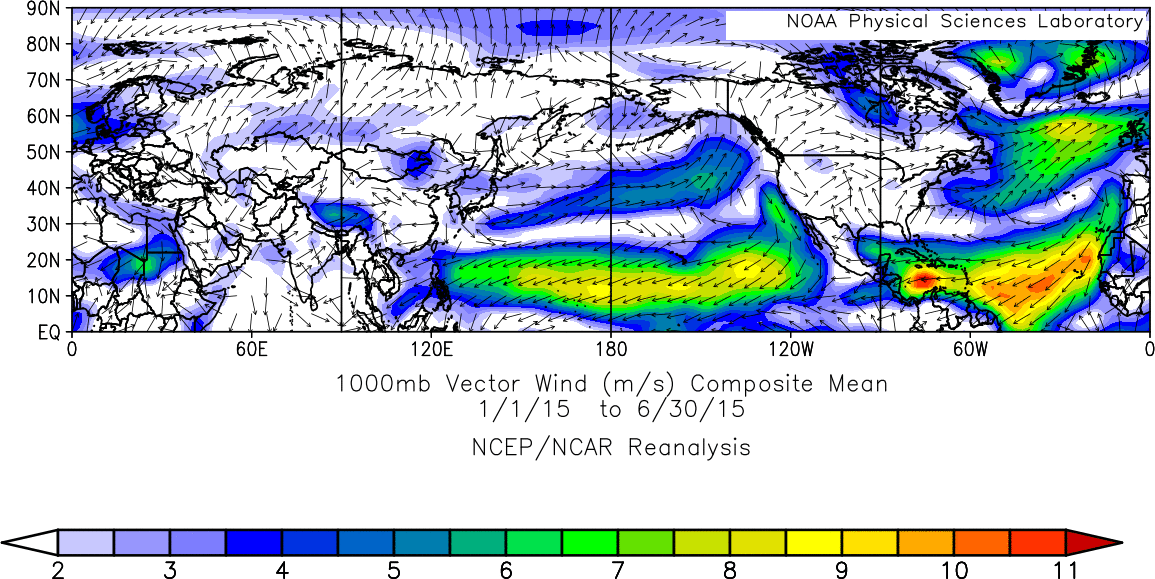
**

**
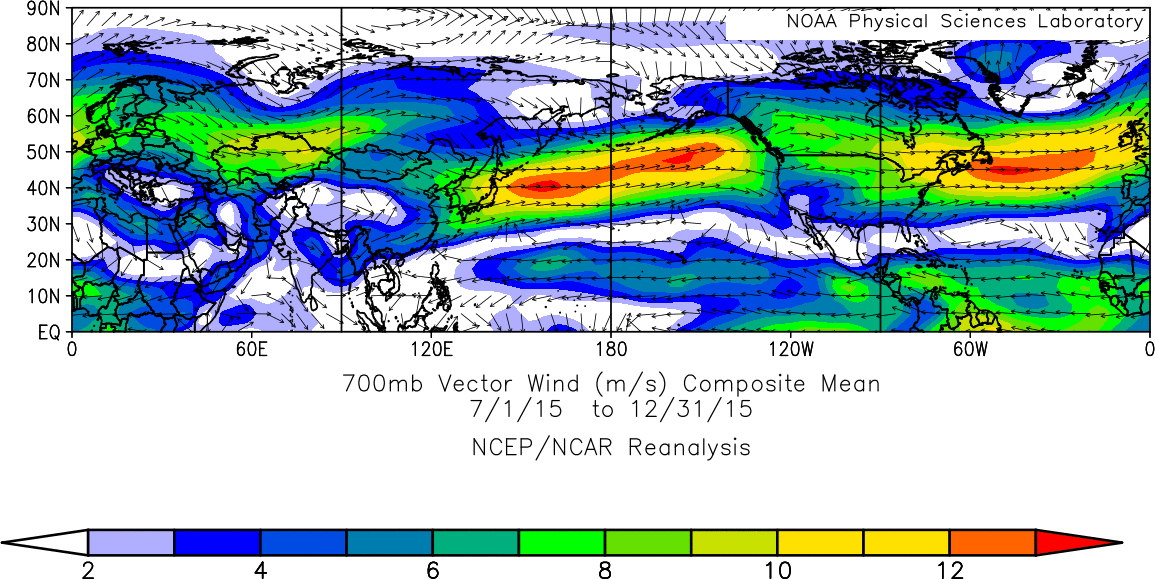

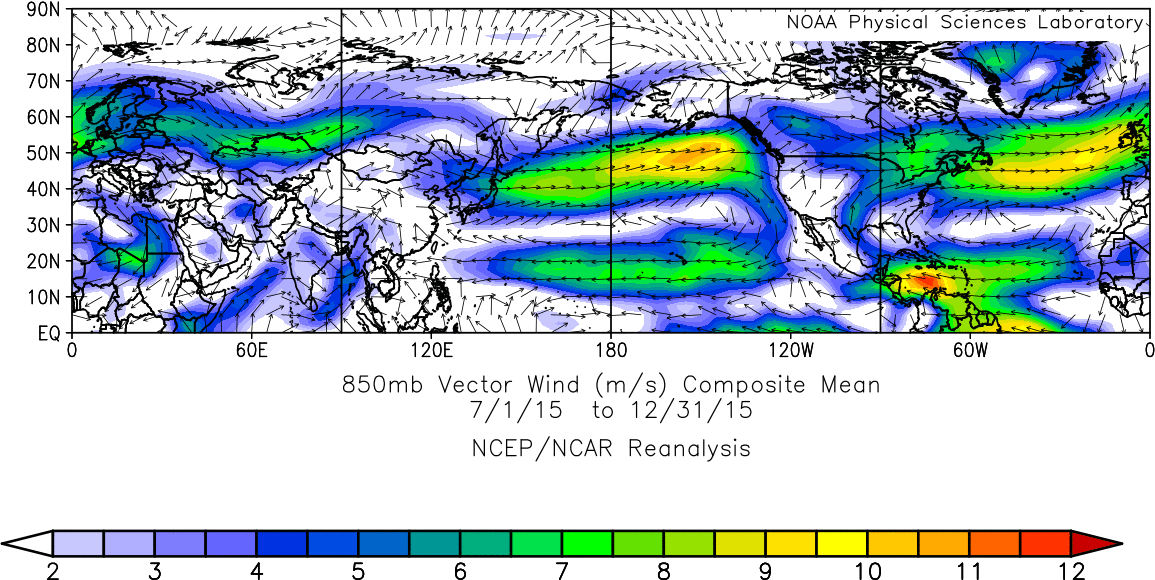

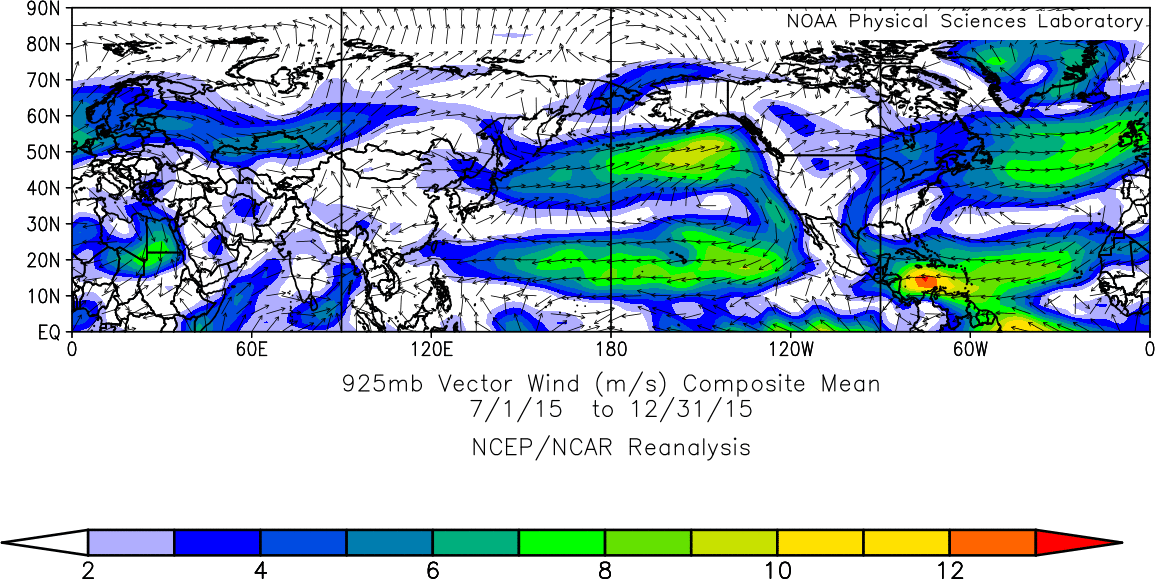

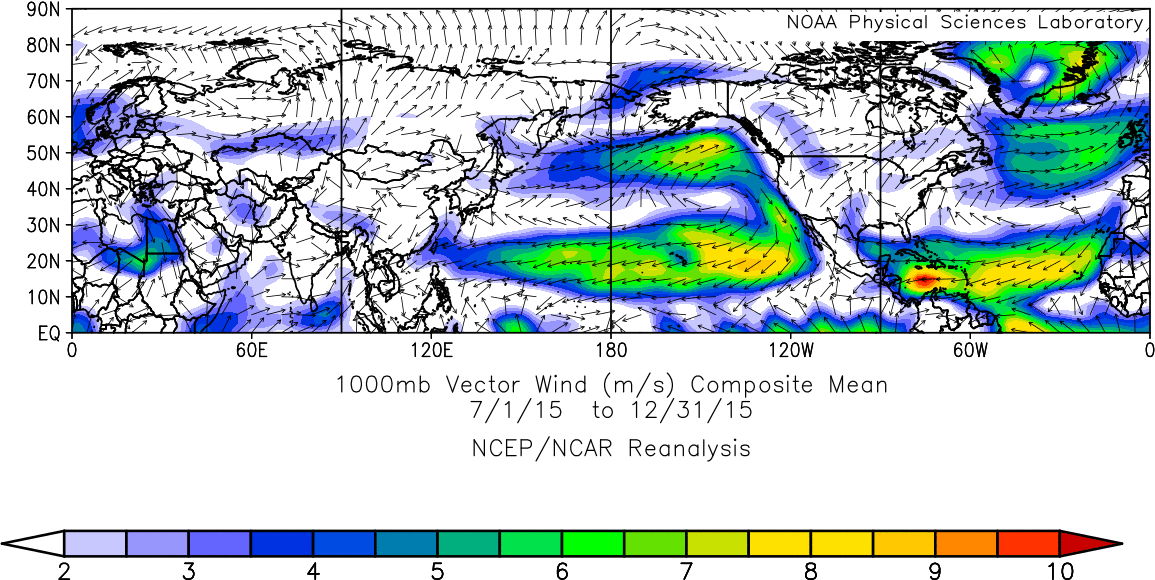
**

**References**

1 Barkan, E. & Luz, B. High‐precision measurements of ^17^O/^16^O and ^18^O/^16^O ratios in CO_2_. *Rapid communications in mass spectrometry* **26**, 2733-2738, doi:10.1002/rcm.6400 (2012).

2 Hofmann, M. E., Horváth, B. & Pack, A. Triple oxygen isotope equilibrium fractionation between carbon dioxide and water. *Earth and Planetary Science Letters* **319**, 159-164, doi:10.1016/j.epsl.2011.12.026 (2012).

3 Welp, L. R. *et al.* Interannual variability in the oxygen isotopes of atmospheric CO_2_ driven by El Niño. *Nature* **477**, 579, doi:10.1038/nature10421 (2011).

4 Liang, M.-C., Mahata, S., Laskar, A. H., Thiemens, M. H. & Newman, S. Oxygen isotope anomaly in tropospheric CO_2_ and implications for CO_2_ residence time in the atmosphere and gross primary productivity. *Scientific Reports* **7**, 13180, doi:10.1038/s41598-017-12774-w (2017).

5 Ciais, P. *et al.* A three‐dimensional synthesis study of δ^18^O in atmospheric CO_2_: 1. Surface fluxes. *Journal of Geophysical Research: Atmospheres* **102**, 5857-5872 (1997).

6 Cuntz, M., Ciais, P., Hoffmann, G. & Knorr, W. A comprehensive global three‐dimensional model of δ^18^O in atmospheric CO_2_: 1. Validation of surface processes. *Journal of Geophysical Research: Atmospheres* **108** (2003).

7 Francey, R. J. & Tans, P. P. Latitudinal variation in oxygen-18 of atmospheric CO_2_. *Nature* **327**, 495 (1987).

8 Stern, L. A., Amundson, R. & Baisden, W. T. Influence of soils on oxygen isotope ratio of atmospheric CO_2_. *Global Biogeochemical Cycles* **15**, 753-759, doi:Doi 10.1029/2000gb001373 (2001).

9 Wingate, L. *et al.* The impact of soil microorganisms on the global budget of δ^18^O in atmospheric CO_2_. *Proceedings of the National Academy of Sciences* **106**, 22411-22415, doi:10.1073/pnas.0905210106 (2009).

10 Farquhar, G. D. *et al.* Vegetation effects on the isotope composition of oxygen in atmospheric CO_2_. *Nature* **363**, 439 (1993).

11 Gillon, J. & Yakir, D. Influence of carbonic anhydrase activity in terrestrial vegetation on the ^18^O content of atmospheric CO_2_. *Science* **291**, 2584-2587, doi:10.1126/science.1056374 (2001).

12 Landais, A., Barkan, E., Yakir, D. & Luz, B. The triple isotopic composition of oxygen in leaf water. *Geochimica et cosmochimica acta* **70**, 4105-4115, doi:10.1016/j.gca.2006.06.1545 (2006).

13 Dai, A. Recent climatology, variability, and trends in global surface humidity. *Journal of Climate* **19**, 3589-3606, doi:Doi 10.1175/Jcli3816.1 (2006).

14 Lal, D. Characteristics of global tropospheric mixing based on man‐made C^14^, H^3^, and Sr^90^. *Journal of Geophysical Research* **71**, 2865-2874, doi:DOI 10.1029/JZ071i012p02865 (1966).

15 Jacob, D. J., Prather, M. J., Wofsy, S. C. & McElroy, M. B. Atmospheric distribution of ^85^Kr simulated with a general circulation model. *Journal of Geophysical Research: Atmospheres* **92**, 6614-6626 (1987).

16 Lawrence, D. M. *et al.* Parameterization improvements and functional and structural advances in version 4 of the Community Land Model. *Journal of Advances in Modeling Earth Systems* **3**, doi:Artn M03001

10.1029/2011ms000045 (2011).

17 IPCC. *Climate Change 2013: The Physical Science Basis. Contribution of Working Group I to the Fifth Assessment Report of IPCC the Intergovernmental Panel on Climate Change*. (Cambridge University Press Cambridge, 2014).

18 Piao, S. *et al.* Evaluation of terrestrial carbon cycle models for their response to climate variability and to CO_2_ trends. *Global change biology* **19**, 2117-2132, doi:10.1111/gcb.12187 (2013).

19 Hoag, K., Still, C., Fung, I. & Boering, K. Triple oxygen isotope composition of tropospheric carbon dioxide as a tracer of terrestrial gross carbon fluxes. *Geophysical Research Letters* **32**, doi:Artn L02802

10.1029/2004gl021011 (2005).

20 Škerlak, B., Sprenger, M. & Wernli, H. A global climatology of stratosphere-troposphere exchange using the ERA-Interim data set from 1979 to 2011. *Atmospheric Chemistry and Physics* **14**, 913-937 (2014).

21 Holton, J. R. *et al.* Stratosphere‐troposphere exchange. *Reviews of geophysics* **33**, 403-439, doi:Doi 10.1029/95rg02097 (1995).

22 Hofmann, M. *et al.* Atmospheric measurements of Δ^17^O in CO_2_ in Göttingen, Germany reveal a seasonal cycle driven by biospheric uptake. *Geochimica et Cosmochimica Acta* **199**, 143-163 (2017).

23 Pearcy, R. & Ehleringer, J. Comparative ecophysiology of C_3_ and C_4_ plants. *Plant, Cell & Environment* **7**, 1-13 (1984).

24 Cousins, A. B., Badger, M. R. & von Caemmerer, S. C_4_ photosynthetic isotope exchange in NAD-ME-and NADP-ME-type grasses. *Journal of Experimental Botany* **59**, 1695-1703 (2008).

25 Luz, B., Barkan, E., Bender, M. L., Thiemens, M. H. & Boering, K. A. Triple-isotope composition of atmospheric oxygen as a tracer of biosphere productivity. *Nature* **400**, 547, doi:Doi 10.1038/22987 (1999).

26 Luz, B. & Barkan, E. Proper estimation of marine gross O_2_ production with ^17^O/^16^O and ^18^O/^16^O ratios of dissolved O_2_. *Geophysical research letters* **38** (2011).

27 Liang, M. C., Blake, G. A. & Yung, Y. L. Seasonal cycle of C^16^O^16^O, C^16^O^17^O, and C^16^O^18^O in the middle atmosphere: Implications for mesospheric dynamics and biogeochemical sources and sinks of CO_2_. *Journal of Geophysical Research: Atmospheres* **113** (2008).

28 Field, C. B., Behrenfeld, M. J., Randerson, J. T. & Falkowski, P. Primary production of the biosphere: integrating terrestrial and oceanic components. *Science* **281**, 237-240, doi:10.1126/science.281.5374.237 (1998).

29 Mahata, S., Bhattacharya, S. & Liang, M. C. An improved method of high‐precision determination of Δ^17^O of CO_2_ by catalyzed exchange with O_2_ using hot platinum. *Rapid Communications in Mass Spectrometry* **30**, 119-131 (2016).

30 Barkan, E., Musan, I. & Luz, B. High‐precision measurements of δ^17^O and ^17^O_excess_ of NBS19 and NBS18. *Rapid Communications in Mass Spectrometry* **29**, 2219-2224 (2015).

31 Liang, M.-C. & Mahata, S. Oxygen anomaly in near surface carbon dioxide reveals deep stratospheric intrusion. *Scientific reports* **5**, 11352, doi:10.1038/srep11352 (2015).

32 Kalnay, E. coauthors, The NCEP/NCAR Reanalysis project. *Bulletin of the American meteorological Society* **77**, 437-471 (1996).
